# Bioinspired elastomer composites with programmed mechanical and electrical anisotropies

**DOI:** 10.1038/s41467-022-28185-z

**Published:** 2022-01-26

**Authors:** Yun Ling, Wenbo Pang, Jianxing Liu, Margaret Page, Yadong Xu, Ganggang Zhao, David Stalla, Jingwei Xie, Yihui Zhang, Zheng Yan

**Affiliations:** 1 Department of Mechanical and Aerospace Engineering, University of Missouri, Columbia, MO 65211 USA; 2 Applied Mechanics Laboratory, Department of Engineering Mechanics, Tsinghua University, 100084 Beijing, China; 3 Center for Flexible Electronics Technology, Tsinghua University, 100084 Beijing, China; 4 Department of Biomedical, Biological and Chemical Engineering, University of Missouri, Columbia, MO 65211 USA; 5 Electron Microscopy Core, University of Missouri, Columbia, MO 65211 USA; 6 Department of Surgery-Transplant and Mary and Dick Holland Regenerative Medicine Program, College of Medicine, University of Nebraska Medical Center, Omaha, NE 68130 USA

**Keywords:** Electronic devices, Polymers, Bioinspired materials

## Abstract

Concepts that draw inspiration from soft biological tissues have enabled significant advances in creating artificial materials for a range of applications, such as dry adhesives, tissue engineering, biointegrated electronics, artificial muscles, and soft robots. Many biological tissues, represented by muscles, exhibit directionally dependent mechanical and electrical properties. However, equipping synthetic materials with tissue-like mechanical and electrical anisotropies remains challenging. Here, we present the bioinspired concepts, design principles, numerical modeling, and experimental demonstrations of soft elastomer composites with programmed mechanical and electrical anisotropies, as well as their integrations with active functionalities. Mechanically assembled, 3D structures of polyimide serve as skeletons to offer anisotropic, nonlinear mechanical properties, and crumpled conductive surfaces provide anisotropic electrical properties, which can be used to construct bioelectronic devices. Finite element analyses quantitatively capture the key aspects that govern mechanical anisotropies of elastomer composites, providing a powerful design tool. Incorporation of 3D skeletons of thermally responsive polycaprolactone into elastomer composites allows development of an active artificial material that can mimic adaptive mechanical behaviors of skeleton muscles at relaxation and contraction states. Furthermore, the fabrication process of anisotropic elastomer composites is compatible with dielectric elastomer actuators, indicating potential applications in humanoid artificial muscles and soft robots.

## Abstract (web summary)

Many biological tissues exhibit directionally dependent properties. Here, authors develop tissue-like elastomer composites with programmed mechanical and electrical anisotropy and discuss potential applications in bioelectronics and humanoid artificial muscles.

## Introduction

Nature has produced biological materials with many intriguing properties and functions that surpass their synthetic counterparts for living organisms to survive and thrive in challenging environments^1–3^. In recent decades, biological materials have provided endless inspiration for developing artificial materials with unique biomimetic properties for a broad spectrum of applications^4–12^. Two exemplary examples are dry adhesives with gecko-inspired hierarchical structures^11^ and epidermal electronics with skin-like compliance^12^. Many biological tissues, represented by muscles, exhibit directionally dependent mechanical and electrical properties, rooted from undulated, preferentially oriented fibrous structures (along the direction of muscle contraction), which play significant roles in preventing tissue damage and regulating bioelectrical signals transmission^13–16^. In this context, synthetic soft materials, which can mimic biological anisotropies, could find promising applications in bioelectronics, artificial muscles, humanoid robots, tissue engineering, and many others.

Recently, a number of artificial soft materials have been developed to offer biomimetic, nonlinear mechanical properties (e.g., J-shaped stress-strain responses)^17–24^. The existing strategies mainly include fundamental molecular engineering of elastomers^17,18^ and ingenious structural designs of networked skeletons of elastomer composites^19–24^. As compared to molecular engineering, structural designs, such as two-dimensional (2D) and three-dimensional (3D) designs of filamentary networks, provide a simple, scalable, easily customizable route to artificial soft materials with tailored nonlinear mechanics. Despite recent significant progress, some challenges remain. Firstly, no strategies have been reported to simultaneously achieve programmed mechanical and electrical anisotropies in artificial materials. Secondly, muscles usually exhibit distinct nonlinear mechanical responses at passive (relaxation) and active (contraction) states^25,26^. The artificial material that can mimic this adaptive property is still lacking.

To bridge the abovementioned gaps, in this work, we introduce the design concept, fabrication techniques, and quantitative design methods to enable bioinspired soft elastomer composites with anisotropic mechanical and electrical properties by leveraging our recent progress in theoretical design-based, mechanically guided 3D assembly^27–32^. Briefly stated, the mechanically guided 3D assembly exploits the stress release in a prestretched elastomer substrate that can self-assemble predesigned 2D patterns into predetermined 3D structures through coordinated translational and rotational geometrical deformations. This approach is applicable to a wide range of materials (e.g., inorganic semiconductors, metals, plastics, 2D materials) and can find broad applications in bioelectronics, 3D electronics, energy harvesting, reconfigurable antennas, and others^33–38^. However, its application in creating bioinspired elastomer composites with mechanical anisotropies is still lacking. Besides, in this work, the stretching-relaxation-induced surface crumpled structures^39,40^ are used to offer electrical anisotropies. Here, both mechanical and electrical anisotropies of the developed elastomer composites can be tailored in a deterministic and independent manner. In addition to theoretical calculations and experimental realizations of anisotropic elastomer composites, their integrations with several active functionalities are also demonstrated. The demonstration examples include biointegrated electrophysiological sensors and electrical stimulators, mimicking of adaptive mechanical responses of skeleton muscles at active and passive states, and integrations with dielectric elastomer actuators, indicating their potential applications in biointegrated electronics and biomimetic artificial muscles.

## Results

### Biomimetic designs of elastomer composites with mechanical and electrical anisotropies

**Fig. 1:**
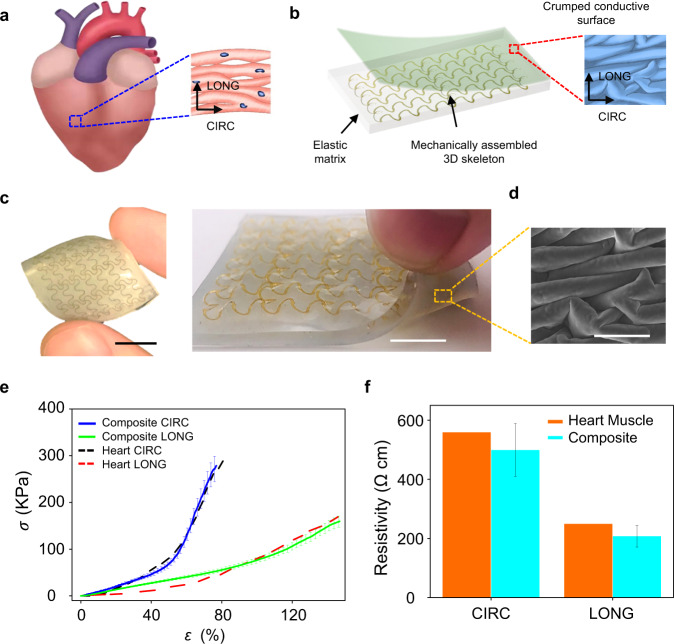
Biomimetic designs of elastomer composites with mechanical and electrical anisotropies. **a** A schematic diagram of right ventricular myocardium with undulated, preferentially oriented cardiac muscle fibers, showing anisotropic mechanical and electrical properties. Arrows indicate anatomically defined circumferential (CIRC) and longitudinal (LONG) axes. **b** Schematic illustrations of elastic matrices embedded with mechanically assembled 3D skeletons and integrated with crumpled conductive surfaces. **c** Optical images of the obtained elastomer composites with crumpled conductive surfaces on the bottom side. Scale bars: 1 cm. **d** SEM image of crumpled conductive surface coated with PEDOT: PSS. Scale bar: 50 µm. **e** Anisotropic, J-shaped stress (*σ*)-strain (*ɛ*) curves of rat right ventricular myocardium along with the circumferential and longitudinal directions, indicating good alignments with the results measured from the developed anisotropic elastomer composites. **f** Comparisons of the electrical resistivity between ventricular myocardium and crumpled conductive surface (the one shown in **d**) along the circumferential and longitudinal directions. Error bars in **e** and **f** are determined from three samples.

In Fig. 1, we present the design concept by using the example of creating elastomer composites that can quantitively mimic the anisotropic mechanical and electrical properties of heart muscle tissue (ventricular myocardium). As shown in Fig. 1a and Supplementary Fig. 1, preferentially oriented cardiac muscle cells bundled within undulated perimysial collagen fibers can yield directionally dependent electrical and mechanical properties along the circumferential (CIRC) and longitudinal (LONG) axes. Besides, upon uniaxial stretching, the tissue exhibits J-shaped stress-strain responses, because undulated collagen fibers first unravel along the loading direction at the initial stage and then begin to straighten. As demonstrated in Fig. 1b, c, our bioinspired design concept incorporates mechanically assembled 3D skeletons of polyimide (PI; elastic modulus: ~2.5 GPa) in soft elastic matrices (silicone; elastic modulus: ~60–330 KPa, depending on material selections) that are integrated with engineered crumpled conductive surfaces. The material fabrication process is illustrated in Supplementary Fig. 2 and described in the Methods section. Here, mechanically assembled 3D skeletons are formed with open-mesh, filamentary network structures that are designed with the guidance of finite element analyses (FEA). Analogous to undulated collagen fibers of biological tissues, 3D skeletons first undergo bending/twisting coupled deformations upon uniaxial tension, and then experience a transition into the stretching-dominated deformation when skeletons along the loading direction are almost straightened. In addition, crumpled conductive layers coated with poly(3,4-ethylenedioxythiophene) polystyrene sulfonate (PEDOT:PSS) and/or silver nanowires (AgNWs) are formed on the surface of the elastomer composite by the relaxation of biaxial prestrains. The electrical anisotropies can be tailored by tuning the amplitudes of biaxial prestrain components. Promisingly, the fabricated elastomer composite with rationally designed 3D skeleton and engineered crumped conductive surface can quantitively match the anisotropic mechanical^14^ (Fig. 1e) and electrical^16^ (Fig. 1f) properties of heart tissues, indicating their potential applications in heart-integrated implants and bioelectronics. Also, the obtained composite materials show outstanding endurance. Both mechanical and electrical properties maintain well after cyclic tests for 500 cycles (Supplementary Fig. 3). The detailed design rules, anisotropy tailoring, FEA simulations, and integrations with several active functionalities are described as follows.

### Deterministically tailored, nonlinear mechanical responses of elastomer composites based on mechanically assembled 3D skeletons

**Fig. 2:**
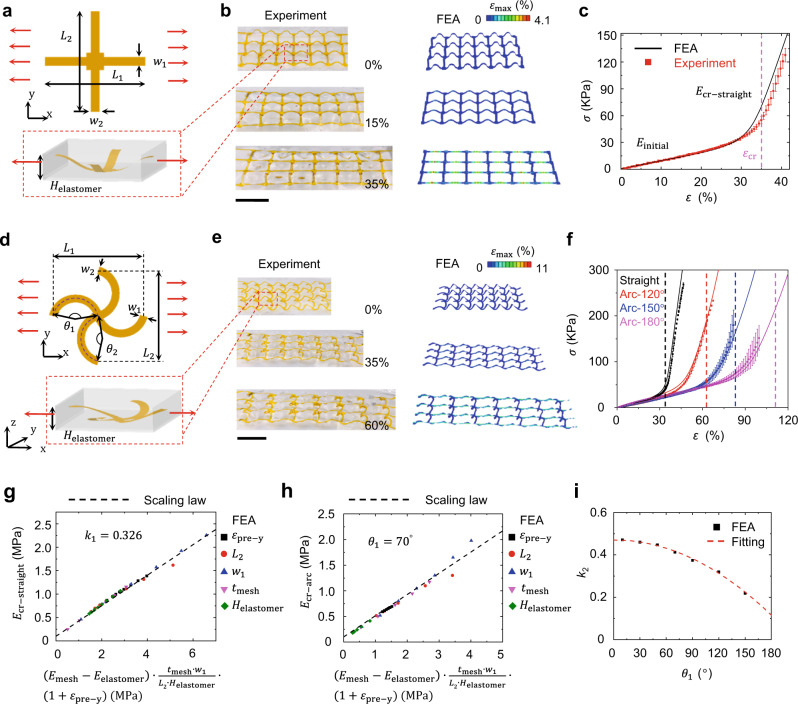
Nonlinear mechanical responses and basic design rules of elastomer composites based on mechanically assembled 3D skeletons. **a** Schematic illustrations of geometric parameters associated with the unit cell of 2D precursor, 3D skeleton, and elastomer encapsulation for the straight-line design. **b** Optical images of one straight-line-based composite and corresponding finite element analysis (FEA) simulations upon uniaxial stretching along the x-axis. Scale bar: 1 cm. **c** Experimental and FEA results of J-shaped stress (*σ*)-strain (*ɛ*) curves for one straight-line-based composite. **d** Scheme of geometric parameters associated with the unit cell of 2D precursor, 3D skeleton, and elastomer encapsulation for the arc-line design. **e** Optical images of one arc-line-based composite and corresponding FEA results with maximum principal strain distributions of 3D skeleton upon uniaxial stretching (arc angle: 120°) along the *x*-axis. Scale bar: 1 cm. **f** Experimental and FEA results of stress-strain curves for one straight-line design and three arc-line designs with different arc angles. Black, red, blue, and pink colour lines refer to the straight-line design and three arc-line designs with arc angles of 120°, 150°, and 180°, respectively. Dash lines refer to critical strains (35%, 63%, 83%, and 112% for the black, red, blue and pink lines, respectively) for the four curves. **g** The scaling law of ${E}_{{{{{{\rm{cr}}}}}}-{{{{{\rm{straight}}}}}}}$ versus $\left({E}_{{{{{{\rm{mesh}}}}}}}-{E}_{{{{{{\rm{e}}}}}}{{{{{\rm{lastomer}}}}}}}\right)\cdot \frac{{t}_{{{{{{\rm{mesh}}}}}}}\cdot {w}_{1}}{{L}_{2}{\cdot H}_{{{{{{\rm{elastomer}}}}}}}}\cdot (1+{\varepsilon }_{{{{{{\rm{pre}}}}}}-{{{{{\rm{y}}}}}}})$ for the straight-line design. **h** The scaling law of ${E}_{{{{{{\rm{cr}}}}}}-{{{{{\rm{arc}}}}}}}$ versus $\left({E}_{{{{{{\rm{mesh}}}}}}}-{E}_{{{{{{\rm{elastomer}}}}}}}\right)\cdot \frac{{t}_{{{{{{\rm{mesh}}}}}}}\cdot {w}_{1}}{{L}_{2}{\cdot H}_{{{{{{\rm{elastomer}}}}}}}}\cdot (1+{\varepsilon }_{{{{{{\rm{pre}}}}}}-{{{{{\rm{y}}}}}}})$ for the arc-line design with ${\theta }_{1}=$70°. **i** The relationship between the parameters ${k}_{2}$ and ${\theta }_{1}$ from FEA results and fitting. The color bars in FEA results represent maximum principal strain distributions of 3D skeletons. Here, ${w}_{1}$, ${w}_{2}$, ${L}_{1}$, ${L}_{2}$ ${H}_{{{{{{\rm{elastomer}}}}}}}$, ${\theta }_{1}$, ${\theta }_{2}$, ${E}_{{{{{{\rm{mesh}}}}}}}$, ${E}_{{{{{{\rm{elastomer}}}}}}}$, ${t}_{{{{{{\rm{mesh}}}}}}}$, ${\varepsilon }_{{{{{{\rm{pre}}}}}}-{{{{{\rm{y}}}}}}}$, ${E}_{{{{{{\rm{cr}}}}}}-{{{{{\rm{straight}}}}}}}$, ${E}_{{{{{{\rm{cr}}}}}}-{{{{{\rm{arc}}}}}}}$, ${k}_{1}$, and ${k}_{2}$ denote the ribbon widths along x and y directions, lengths of unit cells along x and y directions, thickness of elastomer composites, arc angles along x and y directions, elastic moduli of skeleton materials and encapsulation elastomers, thickness of skeleton ribbons, prestrain along y-direction used for 3D assembly, tangential moduli at critical strains for straight-line and arc-line designs, and fitting parameters of straight-line and arc-line designs, respectively. Error bars in **c** and **f** are determined from three samples.

As demonstrated in Supplementary Fig. 4, tissue-like J-shaped nonlinear mechanical responses can be simply divided into three major stages. Stage I is defined as starting from the initial point and ending with the strain where the nonlinear stress-strain relationship occurs. Stage II, i.e., the transition stage, starts from the end of Stage I to the critical strain where the skeletons are straightened along the loading direction. Stage III is defined as starting from the critical strain to the end. Different biological tissues (e.g., skin, heart, and gut) exhibit distinct J-shaped mechanical responses^18^. To satisfy the potential needs of developing various biomimetic materials for different applications, we exploit the combined FEA calculations and experimental validations to study the design rules of elastomer composites to offer deterministically tailored, nonlinear, J-shaped stress-strain responses (Fig. 2 and Supplementary Figs. 5–14). In specific, two types of 3D skeleton designs, straight-line and arc-line designs, are mainly investigated in this work (Supplementary Fig. 5).

Figure 2a shows the basic unit cell of 3D skeletons with the straight-line design. Here, ${w}_{1}$, ${w}_{2}$, ${L}_{1}$, ${L}_{2}$ and *H*_elastomer_ denote the widths of ribbons along x and y directions, lengths of the unit cell along x and y directions, and thickness of the elastomer composite. To study and predict the mechanical property of the elastomer composite, the FEA calculations with periodic boundary conditions (PBC) are conducted (see Methods section). Figure 2b and Supplementary Fig. 6 provide the optical images and FEA simulations of the straight-line-based elastomer composite upon uniaxial stretching (from top to bottom: 0%, 15%, and 35%). Figure 2c shows experimental and FEA results of J-shaped stress-strain curves of this elastomer composite. In this case, Ecoflex 00–20 (elastic modulus: ~60 KPa) is used as the assembly substrate and encapsulation material, and an equal biaxial prestrain of 35% is used to assemble 3D PI skeletons (PI thickness: 25 µm; ribbon widths in the *x*- and *y*-axis: 0.7 mm). In Fig. 2c, ${\varepsilon }_{{{{{{\rm{cr}}}}}}}$ refers to the critical strain, ${E}_{{{{{{\rm{initial}}}}}}}$ refers to the elastic modulus at the initial stage of stretching, and ${E}_{{{{{{\rm{cr}}}}}}-{{{{{\rm{straight}}}}}}}$ represents the tangential modulus at the critical strain. For this composite, the initial tangential modulus is ~60 KPa and the tangential modulus at the critical strain (~35%) is ~900 KPa, indicating ~15 times strain-induced stiffening effects. The FEA simulations with PBC and experimental results agree well with each other, indicating that the FEA with PBC can be exploited as a design tool for developing elastomer composites with programmed nonlinear mechanical properties.

Furthermore, three key parameters (i.e., ${\varepsilon }_{{{{{{\rm{cr}}}}}}}$, ${E}_{{{{{{\rm{cr}}}}}}-{{{{{\rm{straight}}}}}}}$ and ${E}_{{{{{{\rm{initial}}}}}}}$) that characterize J-shaped mechanical responses can be deterministically tailored by judicious designs of 3D skeletons and appropriate material selections of elastic matrices (Supplementary Fig. 7 and Fig. 2g). Specifically, ${E}_{{{{{{\rm{initial}}}}}}}$ and ${\varepsilon }_{{{{{{\rm{cr}}}}}}}$ are mainly controlled by the elastic modulus of the encapsulation material (Supplementary Fig. 7a) and *x*-axis prestrain used for 3D assembly (Supplementary Fig. 7b), respectively. ${E}_{{{{{{\rm{cr}}}}}}-{{{{{\rm{straight}}}}}}}$ can be determined from the equation: ${E}_{{{{{{\rm{cr}}}}}}-{{{{{\rm{straight}}}}}}}={k}_{1}\cdot \left({E}_{{{{{{\rm{mesh}}}}}}}-{E}_{{{{{{\rm{elastomer}}}}}}}\right)\cdot \frac{{t}_{{{{{{\rm{mesh}}}}}}}\cdot {w}_{1}}{{L}_{2}\cdot {H}_{{{{{{\rm{elastomer}}}}}}}}\cdot (1+{\varepsilon }_{{{{{{\rm{pre}}}}}}-{{{{{\rm{y}}}}}}})+{E}_{{{{{{\rm{elastomer}}}}}}}$. Here, ${E}_{{{{{{\rm{mesh}}}}}}}$, ${E}_{{{{{{\rm{elastomer}}}}}}}$, ${t}_{{{{{{\rm{mesh}}}}}}}$ and ${\varepsilon }_{{{{{{\rm{pre}}}}}}-{{{{{\rm{y}}}}}}}$ denote the elastic modulus of the skeleton material, the elastic modulus of the encapsulation elastomer, the thickness of skeleton ribbon, and prestrain along the *y*-axis used for 3D assembly, respectively. Figure 2g demonstrates the scaling law of ${E}_{{{{{{\rm{cr}}}}}}-{{{{{\rm{straight}}}}}}}$ versus $({E}_{{{{{{\rm{mesh}}}}}}}-{E}_{{{{{{\rm{elastomer}}}}}}})\cdot \frac{{t}_{{{{{{\rm{mesh}}}}}}}\cdot {w}_{1}}{{L}_{2}\cdot {H}_{{{{{{\rm{elastomer}}}}}}}}\cdot (1+{\varepsilon }_{{{{{{\rm{pre}}}}}}-{{{{{\rm{y}}}}}}})$ obtained by FEA simulations with PBC for a wide range of ${t}_{{{{{{\rm{mesh}}}}}}}$, ${w}_{1}$, ${\varepsilon }_{{{{{{\rm{pre}}}}}}-{{{{{\rm{y}}}}}}}$, ${L}_{2}$ and ${H}_{{{{{{\rm{elastomer}}}}}}}$. The parameter ${k}_{1}$ is obtained as 0.326. According to these analyses, the J-shaped nonlinear mechanical response of the elastomer composite can be accurately designed to match those of various biological tissues. In this study, a series of experiments and FEA simulations are conducted by adjusting the ribbon widths (Supplementary Fig. 7c) and prestrains in the *y*-axis used for 3D assembly (Supplementary Fig. 7d).

J-shaped stress-strain curves of biological tissues usually exhibit a moderate transition part^18^, which cannot be well achieved based on straight-line designs. To address this issue, we further study arc-line-based 3D skeleton designs to provide additional degrees of tunability. Figure 2d shows the unit cell of the arc-line design associated with six geometrical parameters: ${L}_{1}$, ${L}_{2}$, ${w}_{1}$, ${w}_{2}$, *H*_elastomer_ and arc angles (${\theta }_{1}$ and ${\theta }_{2}$) in the *x*-axis and *y*-axis. For simplicity, in the designs studied in this paper, ${\theta }_{1}$ is equal to ${\theta }_{2}$. Figure 2e and Supplementary Fig. 8 provide optical images and FEA simulations for an arc-line-based elastomer composite upon stretching (from top to bottom: 0%, 35%, and 60%; arc angle: 120°). Figure 2f provides the experimental and FEA results of J-shaped stress-strain curves of elastomer composites based on the straight-line design and arc-line designs with arc angles of 120°, 150°, and 180°. Here, an equal biaxial prestrain of 35% is used to assemble the four 3D PI skeletons with different arc angles for easy comparison (PI thickness: 25 µm; ribbon widths in the *x*- and *y*-axis: 0.7 mm). The arc-line designs can endow the resulting elastomer composites with longer transition parts than the straight-line design, mainly due to the twisting and unwinding of the 3D structures during stretching.

Analogous to straight-line designs, the initial tangential modulus of the arc-line-based elastomer composite is mainly controlled by the elastic modulus of the encapsulation material. And the tangential modulus at the critical strain (${E}_{{{{{{\rm{cr}}}}}}-{{{{{\rm{arc}}}}}}}$) can be determined from the equation: ${E}_{{{{{{\rm{cr}}}}}}-{{{{{\rm{arc}}}}}}}={k}_{2}\cdot ({E}_{{{{{{\rm{mesh}}}}}}}-{E}_{{{{{{\rm{elastomer}}}}}}})\cdot \frac{{t}_{{{{{{\rm{mesh}}}}}}}\cdot {w}_{1}}{{L}_{2}\cdot {H}_{{{{{{\rm{elastomer}}}}}}}}\cdot (1+{\varepsilon }_{{{{{{\rm{pre}}}}}}-{{{{{\rm{y}}}}}}})+{E}_{{{{{{\rm{elastomer}}}}}}}$. Figure 2h and Supplementary Fig. 9 provide the scaling law of ${E}_{{{{{{\rm{cr}}}}}}-{{{{{\rm{arc}}}}}}}$ versus $({E}_{{{{{{\rm{mesh}}}}}}}-{E}_{{{{{{\rm{elastomer}}}}}}})\cdot \frac{{t}_{{{{{{\rm{mesh}}}}}}}\cdot {w}_{1}}{{L}_{2}\cdot {H}_{{{{{{\rm{elastomer}}}}}}}}\cdot (1+{\varepsilon }_{{{{{{\rm{pre}}}}}}-{{{{{\rm{y}}}}}}})$ obtained by FEA for a wide range of ${t}_{{{{{{\rm{mesh}}}}}}}$, ${w}_{1}$, ${\varepsilon }_{{{{{{\rm{pre}}}}}}-{{{{{\rm{y}}}}}}}$, ${L}_{2}$, ${H}_{{{{{{\rm{elastomer}}}}}}}$ and ${\theta }_{1}$. The parameter fitting of ${k}_{2}$, which is related to the arc angles (${\theta }_{1}$), is provided in Fig. 2i. The fitted relationship can be expressed as $k_{2}\left({\theta }_{1}\right)=2.57\times {10}^{-5}\cdot {\theta }_{1}-1.1085\times {10}^{-5}\cdot {{\theta }_{1}}^{2}+0.47$. The critical strain for the arc-line design (${\varepsilon }_{{{{{{\rm{cr}}}}}}-{{{{{\rm{arc}}}}}}}$) is defined as the strain at which the elastomer composite is stretched to the arc length of the arc strip. Here, ${\varepsilon }_{{{{{{\rm{cr}}}}}}-{{{{{\rm{arc}}}}}}}=\pi \cdot ({\varepsilon }_{{{{{{\rm{pre}}}}}}}+1)\kern-0.1pc\cdot\kern-0.1pc {\theta }_{1}/\left[360\cdot {{{{{\rm{sin }}}}}}\left(\frac{{\theta }_{1}}{2}\right)\right]-1$. Increasing the ribbon width (${w}_{1}$) can enhance the coupling between the 3D skeleton and encapsulation material, thereby providing another way to adjust the transition part (Supplementary Fig. 10).

The nonlinear mechanical property of the elastomer composite along the *x*- and *y*-axis can be tuned independently, providing access to anisotropic, nonlinear mechanical properties. For example, as demonstrated in Fig. 1e, the anisotropic nonlinear mechanical property, mimicking that of the ventricular myocardium, is achieved based on our reverse design method. For this composite, the corresponding 2D precursor is shown in Supplementary Fig. 11. Prestrains of ${\varepsilon }_{{{{{{\rm{x}}}}}}}=10 \%$ and ${\varepsilon }_{{{{{{\rm{y}}}}}}}=50 \%$ are used to assemble the 3D PI skeleton because heart tissues demonstrate anisotropic, nonlinear mechanical responses along the CIRC and LONG axes. Ecoflex 0020 is used as the assembly substrate and encapsulation material. Besides, for the aforementioned straight-line and arc-line designs, while the elastomer composite is stretched not along orthogonal directions (e.g., 45° direction), 3D skeletons undergo non-principal direction deformation, which delays the transition of J-shaped curve. In this case, the shear stress concentrates on the corner of the skeleton, which could easily cause the fracture of the skeleton. To address this issue, a modified design (Supplementary Fig. 12a) is exploited. Supplementary Figure 12b shows stress-strain curves under uniaxial stretching along Direction I (*x*-axis) and Direction II (45° direction) for this modified design, and along Direction II for the straight-line design. The FEA simulations agree well with the experimental results, and the stress-strain curve for the modified design along Direction II appears as a J shape (compared with the straight-line design), indicating that the modified design has a good protective effect along 45° direction for the elastomer composite. In addition, with rational designs of multilayered 3D skeletons, more tunablity of nonlinear mechanical responses, such as multiple critical strains and multistage stepped moduli, can be achieved in the resulting elastomer composites (Supplementary Fig. 13). Also, the method demonstrates a broad generality and can be applied to the designs and fabrications of elastomer composites with small-sized 3D skeletons (Supplementary Fig. 14).

### Crumpled anisotropic conductive surfaces

**Fig. 3:**
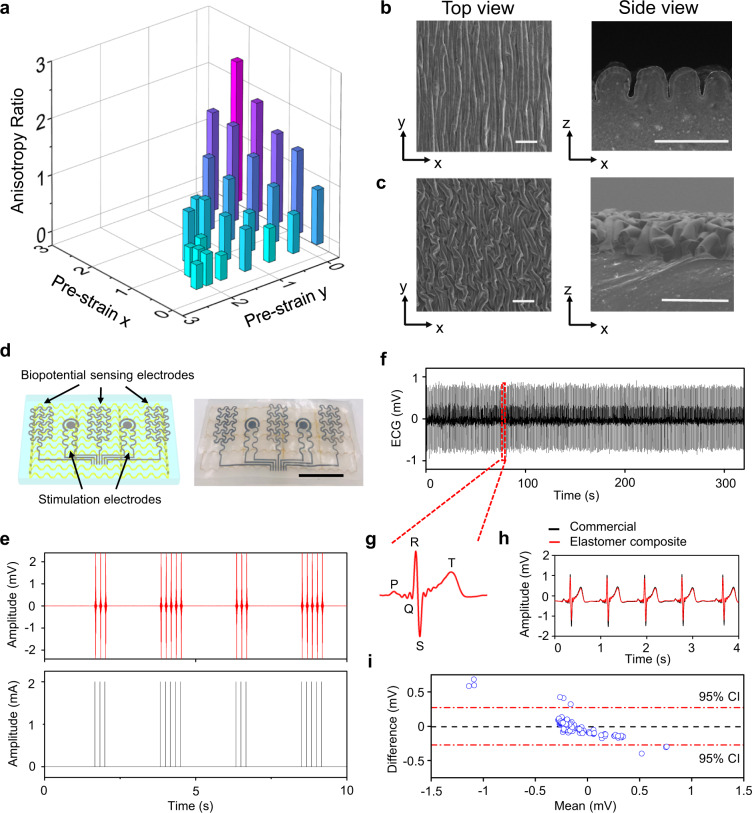
Crumpled anisotropic conductive surfaces. **a** Tunable electrical anisotropies controlled by applied prestrains on the x and y axes. SEM images of crumpled anisotropic conductive surfaces formed by applying prestrains of *ε*_x_ = 100% and *ε*_y_ = 0 (**b**) and *ε*_x_ = 150% and *ε*_y_ = 40% (**c**). Scale bars: 100 µm. Here, PEDOT:PSS is coated as surface conductive layers. **d** Electrophysiological sensors and bioelectrical stimulators based on the anisotropic elastomer composites. Scale bar: 1 cm. **e** EMGs (top) recorded from the related muscle (flexor digitorum superficialis) during programed electrical stimulations (bottom). **f** Continuous ECG recording from the human chest. **g** Magnified ECG signal, indicating clear P-wave, QRS complex, and T-wave. Direct comparisons (**h**) and Bland-Altman analysis with 95% confidence interval (95% CI) (**i**) of ECGs recorded using electrophysiological sensors based on anisotropic elastomer composites (red curve in **h**) and conventional Ag/AgCl gel electrodes (black curve in **h**).

The electrical properties of biological tissues can determine the pathway of the current flow through the body, and are critical in a wide range of biomedical applications^15,16,41^. In addition to mechanical anisotropies, muscle tissues are also highly anisotropic in electrical conductivity because the current flows more easily along muscle fibers than across the fibers. Simultaneous mimicking of these two unique features can blur the boundaries between biological tissues and synthetic materials in both the mechanical and electrical behavior to facilitate the development of next-generation humanoid artificial muscles and biointegrated electronics. As shown in Fig. 3a–c and Supplementary Fig. 15, crumpled surface hierarchical structures are adopted to endow the elastomer composite with desired electrical anisotropies. Here, O_2_ plasma treatment is used to induce a thin stiff surface layer (SiO_2_; ~3 µm in thickness) on the biaxially prestretched elastomer composite (~4 mm in thickness), followed by coating of conductive materials (PEDOT:PSS, AgNWs, or their hybrids; less than 100 nm in thickness). Releasing the prestrain transforms top layers (SiO_2_ plus conductive materials) into crumpled hierarchical structures, due to the mismatch of the elastic properties between top stiff layers and bottom elastic matrices. The geometries of crumpled conductive surfaces can be tailored by regulating applied prestrains, providing a means to tune electrical anisotropies. As shown in Fig. 3a, a wide range of anisotropic resistivity ratios (from 0.4 to 2.5) between the *x*- and *y*-axis, measured by a four-probe method, have been realized. The SEM images of some representative crumbled conductive surfaces formed with different prestrains are shown in Figs. 1d, 2b-c, and Supplementary Fig. 15, where PEDOT:PSS is used as the surface conductive material. In Fig. 1d, prestrains of *ε*_x_ = 40% and *ε*_y_ = 150% are used to form the crumpled conductive surfaces to quantitatively match the anisotropic electrical properties of heart tissue.

Besides, PEDOT:PSS, a mixed ionic and electronic conductor, is suitable for biomimetic materials preparation and biotic-abiotic interfacing, but suffers from relatively limited electrical conductivities^42^. AgNWs exhibit outstanding electrical conductivities but are constrained from poor long-term stability, since they are prone to oxidation in air^43^. To demonstrate potential applications of developed elastomer composites in biointegrated electronics, the hybrid of PEDOT:PSS and AgNWs is used as the surface conductive layer for device fabrication, demonstrating both high electrical conductivity and long-term stability (Supplementary Fig. 16). The prototypical device examples include bioelectrical stimulators and biopotential sensors that are made via spray printing of the hybrid conductor on anisotropic elastomer composites (Fig. 3d). Figure 3e demonstrates that the electromyogram (EMG) signals, recorded from the muscle (flexor carpi radialis) contraction induced by programmed electrical stimulations, align very well with the electrical stimulation signals, indicating the capability of delivering on-demand electrical interventions for potential disease treatment, pain alleviation, and rehabilitation. Also, the electrophysiological sensor can record electrocardiogram (ECG) signals from the human chest in a continuous, high-fidelity way (Fig. 3f). The magnified ECG signal shows a clear P-wave, QRS complex, and T-wave (Fig. 3g). Direct comparisons (Fig. 3h) and Bland-Altman analysis (Fig. 3i) indicate good alignments between ECG signals recorded with electrophysiological sensors based on anisotropic elastomer composites and those measured with conventional silver/silver chloride (Ag/AgCl) gel electrodes.

Besides, oriented conductive fibers have been recently used to guide the cell growth for tissue engineering by providing both topographical and electrical cues^44^. As shown in Fig. 3a-c and Supplementary Fig. 15, the strategy reported in this work can provide fine control of both surface topographies and electrical anisotropies via tailoring applied prestrains. Thus, it holds great promise for applications in tissue engineering, by providing customized, anisotropic surface structures and electrical cues for the guided growth of various cell types. In addition to potential applications in tissue engineering, as discussed in the subsequent section, composite materials with skeleton muscle-like adaptive mechanical behaviors have been developed using our method for potential applications in humanoid artificial muscles and soft robots.

### Biomimetic elastomer composites with electrothermally triggered, adaptive mechanical properties

**Fig. 4:**
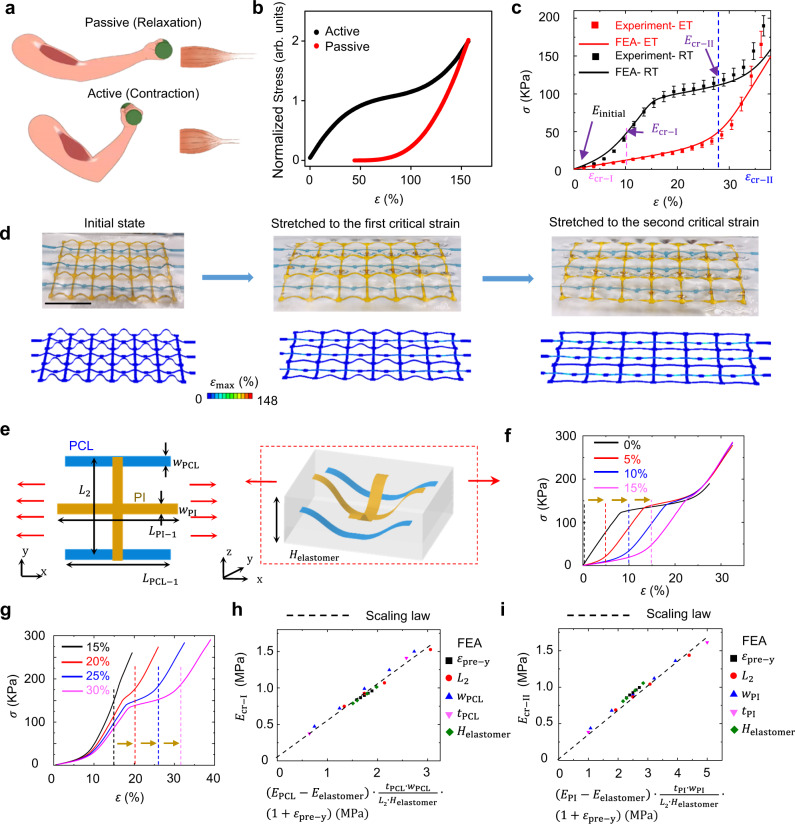
Adaptive nonlinear mechanical responses and design rules of electrothermally responsive, biomimetic elastomer composites. **a** Scheme of human skeleton muscles at passive (relaxation) and active (contraction) states. **b** Schematic stress-strain responses of skeleton muscles at passive and active states. **c** Experimental and finite element analysis (FEA) results of stress (*σ*)-strain (ε) curves of developed electrothermally responsive, elastomer composites at room temperature (RT) and elevated temperature (ET; ~60 °C). Error bars are determined from three samples. **d** Optical images (top) and corresponding FEA simulations (bottom) of the electrothermally responsive composites upon stretching at room temperature. The color bars in FEA results represent maximum principal strain distributions of 3D skeleton. Blue ribbons: polycaprolactone (PCL). Yellow ribbons: polyimide (PI). Scale bar: 1 cm. PCL skeletons are colorized by blue dyes for easy visualization. **e** Schematic illustrations of the geometric parameters associated with the unit cell of 2D precursor, 3D skeleton, and elastomer encapsulation for the electrothermally responsive elastomer composite. **f** FEA results of stress-strain curves for electrothermally responsive elastomer composites with PCL skeletons assembled with different prestrains (0%, 5%, 10%, 15%). The prestrain of assembling PI skeletons is 25%. **g** FEA results of stress-strain curves for electrothermally responsive elastomer composites with PI skeletons assembled with different prestrains (15%, 20%, 25%, 30%). The prestrain of assembling PCL skeletons is 10%. In **f** and **g**, uniaxial prestrains along the *x*-axis are used to assemble 3D skeletons. **h** The scaling law of ${E}_{{{{{{\rm{cr}}}}}}-{{{{{\rm{I}}}}}}}$ versus $\left({E}_{{{{{{\rm{PCL}}}}}}}-{E}_{{{{{{\rm{elastomer}}}}}}}\right)\cdot \frac{{t}_{{{{{{\rm{PCL}}}}}}}\cdot {w}_{{{{{{\rm{PCL}}}}}}}}{{L}_{2}{\cdot H}_{{{{{{\rm{elastomer}}}}}}}}\cdot (1+{\varepsilon }_{{{{{{\rm{pre}}}}}}-{{{{{\rm{y}}}}}}})$ for electrothermally responsive elastomer composites. **i** The scaling law of ${E}_{{{{{{\rm{cr}}}}}}-{{{{{\rm{II}}}}}}}$ versus $\left({E}_{{{{{{\rm{PI}}}}}}}-{E}_{{{{{{\rm{elastomer}}}}}}}\right)\cdot \frac{{t}_{{{{{{\rm{PI}}}}}}}\cdot {w}_{{{{{{\rm{PI}}}}}}}}{{L}_{2}{\cdot H}_{{{{{{\rm{elastomer}}}}}}}}\cdot (1+{\varepsilon }_{{{{{{\rm{pre}}}}}}-{{{{{\rm{y}}}}}}})$ for electrothermally responsive elastomer composites. Here, ${w}_{{{{{{\rm{PI}}}}}}}$, ${w}_{{{{{{\rm{PCL}}}}}}}$, ${t}_{{{{{{\rm{PI}}}}}}}$, ${t}_{{{{{{\rm{PCL}}}}}}}$, ${L}_{{{{{{\rm{PI}}}}}}-1}$, ${L}_{{{{{{\rm{PCL}}}}}}-1}$, ${L}_{2}$, ${H}_{{{{{{\rm{elastomer}}}}}}}$, ${E}_{{{{{{\rm{PCL}}}}}}}$, ${E}_{{{{{{\rm{elastomer}}}}}}}$, ${E}_{{{{{{\rm{cr}}}}}}-{{{{{\rm{I}}}}}}}$, and ${E}_{{{{{{\rm{cr}}}}}}-{{{{{\rm{II}}}}}}}$ denote the widths of PI and PCL ribbons along the *x*-axis, thicknesses of PI and PCL ribbons, the width of PCL ribbon, length of the unit cell of PI ribbon along the *x*-axis, length of the unit cell of PCL ribbon along the *x*-axis, length of the unit cell for both PCL and PI ribbons along the *y*-axis, thickness of the composite, elastic moduli of PCL and encapsulation elastomers, the first critical strain, and the second critical strain, respectively.

As shown in Fig. 4a, b, skeleton muscle exhibits distinct nonlinear mechanical responses along longitudinal directions at passive (relaxation) and active (contraction) states under uniaxial stretching^25,26^. Here, the passive (relaxation) state refers to the muscle at its original state without being activated by nervous or other stimulation. And the active (contraction) state refers to the muscle activated with stimulation, indicating changes in the tension and/or length. In this research, we have developed the electrothermally responsive elastomer composite that can mimic the adaptive mechanical behavior of skeleton muscles. The elastomer composite consists of the hybrid skeletons of polycaprolactone (PCL; melting point: ~60 °C) and PI (glass transition temperature: ~250–340 °C). The PCL and PI skeletons are assembled with uniaxial prestrains of ${\varepsilon }_{{{{{{\rm{x}}}}}}}=12 \%$ and ${\varepsilon }_{{{{{{\rm{x}}}}}}}=28 \%$, respectively. In addition, the composite is integrated with crumped conductive surfaces, coated with hybrids of PEDOT:PSS and AgNWs, as joule-heating elements. Supplementary Figure 17 shows that the joule heater can operate well under stretching. A thermal couple and an IR camera are used to measure the temperatures of the PCL skeletons and composite surfaces, respectively (Supplementary Fig. 18). The stress-strain curves of the obtained composite at room temperature (RT) and elevated temperature (ET; ~60 °C) are provided in Fig. 4c, which can mimic the adaptive mechanical behaviors of skeleton muscles at active and passive states, respectively. Figure 4d and Supplementary Fig. 19 provide optical images and FEA simulations of the composite under different levels of tensile strains (initial state: 0%; first critical strain: 12%; second critical strain: 28%). At the elevated temperature, the composite exhibits typical J-shaped mechanical responses, because PCL skeletons melt and PI skeletons dominate the mechanical property of the composite. Supplementary Figure 20 demonstrates the mechanical behavior of the PCL without patterns and the composite embedded with 3D PCL skeletons. At room temperature, the stress-strain curves of elastomer composites are mainly determined by the first critical strain (${\varepsilon }_{{{{{{\rm{cr}}}}}}-{{{{{\rm{I}}}}}}}$), second critical strain (${\varepsilon }_{{{{{{\rm{cr}}}}}}-{{{{{\rm{II}}}}}}}$), initial tangential modulus $({E}_{{{{{{\rm{initial}}}}}}})$, tangential modulus at the first critical strain (${E}_{{{{{{\rm{cr}}}}}}-{{{{{\rm{I}}}}}}}$), and tangential modulus at the second critical strain (${E}_{{{{{{\rm{cr}}}}}}-{{{{{\rm{II}}}}}}}$). Figure 4e shows the geometric parameters associated with the hybrid PCL/PI skeleton designs. ${E}_{{{{{{\rm{initial}}}}}}}$ relies on the elastic modulus of the encapsulation material. As shown in Fig. 4f-g, ${\varepsilon }_{{{{{{\rm{cr}}}}}}-{{{{{\rm{I}}}}}}}$ and ${\varepsilon }_{{{{{{\rm{cr}}}}}}-{{{{{\rm{II}}}}}}}$ can be adjusted by tuning the prestrains to assemble 3D PCL and PI skeletons, respectively. ${E}_{{{{{{\rm{cr}}}}}}-{{{{{\rm{I}}}}}}}$ can be determined approximately by ${E}_{{{{{{\rm{cr}}}}}}-{{{{{\rm{I}}}}}}}= 0.52\cdot ({E}_{{{{{{\rm{PCL}}}}}}}-{E}_{{{{{{\rm{elastomer}}}}}}})\cdot \frac{{t}_{{{{{{\rm{PCL}}}}}}}\cdot {w}_{{{{{{\rm{PCL}}}}}}}}{{L}_{2}\cdot {H}_{{{{{{\rm{elastomer}}}}}}}}\cdot (1+{\varepsilon }_{{{{{{\rm{pre}}}}}}-{{{{{\rm{y}}}}}}})+{E}_{{{{{{\rm{elastomer}}}}}}}$. Here, ${w}_{{{{{{\rm{PCL}}}}}}}$, ${L}_{{{{{{\rm{PCL}}}}}}-1}$, ${L}_{2}$ and ${E}_{{{{{{\rm{PCL}}}}}}}$ denote the width of PCL ribbon along the *x*-axis, length of PCL unit cell along the *x*-axis, length of the unit cell for both PCL and PI along the *y*-axis, and elastic modulus of PCL, respectively. Figure 4h shows the scaling law of ${E}_{{{{{{{\rm{cr}}}}}}-{{{{{\rm{I}}}}}}}}$ versus $({E}_{{{{{{\rm{PCL}}}}}}}-{E}_{{{{{{\rm{elastomer}}}}}}})\cdot {t}_{{{{{{\rm{PCL}}}}}}}\cdot {w}_{{{{{{\rm{PCL}}}}}}}\cdot (1+{\varepsilon }_{{{{{{{\rm{pre}}}}}}-{{{{{\rm{y}}}}}}}})/({L}_{2}\cdot {H}_{{{{{{\rm{elastomer}}}}}}})$ obtained by FEA. Similarly, ${E}_{{{{{{\rm{cr}}}}}}-{{{{{\rm{II}}}}}}}$ can be determined by ${E}_{{{{{{\rm{cr}}}}}}-1}=0.34\cdot ({E}_{{{{{{\rm{PI}}}}}}}-{E}_{{{{{{\rm{elastomer}}}}}}})\cdot {t}_{{{{{{\rm{PI}}}}}}}\cdot {w}_{{{{{{\rm{PI}}}}}}}\cdot (1+{\varepsilon }_{{{{{{\rm{pre}}}}}}-{{{{{\rm{y}}}}}}})/({L}_{2}\cdot {H}_{{{{{{\rm{elastomer}}}}}}})+{E}_{{{{{{\rm{elastomer}}}}}}}$, as shown by Fig. 4i. Supplementary Figures 21 and 22 provide more results about the tunability of stress-strain curves of the PCL/PI hybrid composites by adjusting other parameters. In addition, while the PCL skeleton is overstretched during the process, it can return to the initial state after releasing the strain at the elevated temperature (~60 °C), as shown in Supplementary Fig. 23.

### Integration with dielectric elastomer actuators

**Fig. 5:**
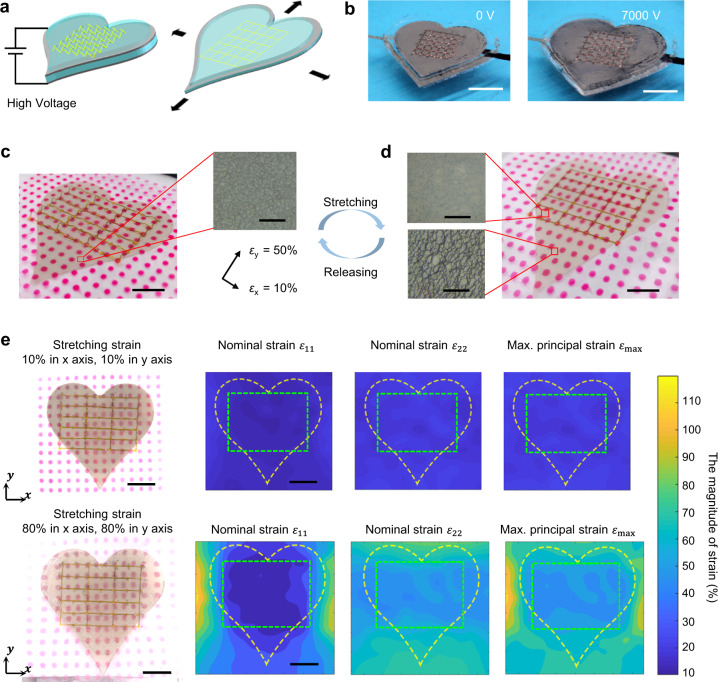
Integration with dielectric elastomer actuators. Schematic illustrations (**a**) and optical images (**b**) of electric field-induced deformations for a heart-like dielectric elastomer actuator integrated with anisotropic elastomer composites. Scale bars: 1 cm. Strain visualization and protection of compliant electrodes of a heart-like dielectric elastomer actuator integrated with anisotropic elastomer composites without stretching (**c**) and with large stretching (**d**; ${\varepsilon }_{{{{{{\rm{x}}}}}}}={\varepsilon }_{{{{{{\rm{y}}}}}}}=80 \%$). The prestrains of ${\varepsilon }_{{{{{{\rm{x}}}}}}}=10 \%$ and ${\varepsilon }_{{{{{{\rm{y}}}}}}}=50 \%$ are used to assemble 3D polyimide (PI) skeletons. Scale bars: 1 cm. Inset optical images indicate the morphologies of the compliant electrodes in different regions without and with stretching, scales bars: 100 μm. **e** The quantitive analysis of nominal strain components (${\varepsilon }_{11}$ and ${\varepsilon }_{22}$) and maximum principal strain (${\varepsilon }_{{{{{{\rm{max }}}}}}}$) under small (${\varepsilon }_{{{{{{\rm{x}}}}}}}={\varepsilon }_{{{{{{\rm{y}}}}}}}=10 \%$; top) and large stretching (${\varepsilon }_{{{{{{\rm{x}}}}}}}={\varepsilon }_{{{{{{\rm{y}}}}}}}=80 \%$; bottom), indicating that skeleton-integrated regions, marked with green dashed lines, exhibit tissue-like nonlinear mechanical responses. Scale bars: 1 cm.

Dielectric elastomer actuators, which use electric fields to deform elastomeric materials sandwiched between two compliant electrodes, have demonstrated promising applications in artificial muscles, soft robots, programmed motions for 3D assembly, and biomedical devices because of their high response speed and low power requirements^32,45–50^. Here, we have demonstrated that anisotropic elastomer composites can be integrated with dielectric elastomer actuators (Fig. 5a), indicating potentials of developing humanoid artificial muscles and soft robots with biomimetic anisotropic properties. Figure 5b, Supplementary Figs. 24–25, and Supplementary Movie 1 show the successful integration of anisotropic elastomer composites with a heart-like dielectric elastomer actuator. Furthermore, well-designed 3D skeletons can endow dielectric elastomer actuators with tissue-like nonlinear mechanical behaviors (Fig. 5c–e, Supplementary Fig. 26, and Supplementary Movies 2-3), i.e., initially soft and stiffening rapidly as they are stretched to a relatively large extent, thereby preventing large deformation-induced damages. For example, the optical images in Fig. 5c-d demonstrates that AgNWs compliant electrodes within the regions of 3D skeletons maintain well after large stretching (*ε*_x_ = *ε*_y_ = 80%). By contrast, cracks form in AgNWs compliant electrodes outside the regions of 3D skeletons. Here, the prestrains of (*ε*_x_ = *ε*_y_ = 50%) are used to form crumpled compliant electrodes of AgNWs. Red dots in the optical images provide the direct strain visualization. Figure 5e shows quantitive analyses of 3D skeleton-enabled nonlinear strain regulations, indicating the distributions of nominal strain components (${\varepsilon }_{11}$ and ${\varepsilon }_{22}$) and maximum principal strain (${\varepsilon }_{{{{{{\rm{max }}}}}}}$) during small stretching (${\varepsilon }_{{{{{{\rm{x}}}}}}}={\varepsilon }_{{{{{{\rm{y}}}}}}}=10 \%$; top panels) and large stretching (${\varepsilon }_{{{{{{\rm{x}}}}}}}={\varepsilon }_{{{{{{\rm{y}}}}}}}=80 \%$; bottom panels). The maximum principal strain is considered as the key factor of material fracture, which can be obtained from three in-plane strain components^51^. As shown in Fig. 5e, skeleton-integrated DEA regions, marked with green dash lines, deform freely when applied strains are below the critical strains (~10% for x direction and ~50% for y direction). When applied strains are above the critical strains, skeleton-integrated regions are protected and indicate the reduced strains as compared to the DEA parts outside skeleton-integrated regions, marked with yellow dash lines. In addition, DEA-based artificial muscles with and without 3D skeletons have been further developed (Supplementary Fig. 26 and Supplementary Movies 2-3). It is notable that high actuating voltages can overstretch the DEA-based artificial muscles, leading to the electrical breakdown and device failure (Supplementary Fig. 26 and Supplementary Movie 2). By contrast, the DEA-based artificial muscles with 3D skeletons exhibit tissue-like nonlinear mechanical behaviors (Supplementary Fig. 26 and Supplementary Movie 3), and can efficiently prevent the device from being overstretched. In brief, at a low actuating voltage (≤5500 V for this study), the actuation-induced strain is below the critical strains (the horizontal direction: ~15% and the vertical direction: ~25% for this design) and the artificial muscle can actuate freely. At a high actuating voltage, the actuation-induced strain is above the critical strains and the artificial muscle stiffens rapidly to avoid the overstretching and device failure.

It is worth noting that in our previous work^32^, the electro-mechanically controlled 3D assembly was presented with the use of dielectric elastomer platforms. The major difference between this work and the previous work are summarized as follows. Firstly, in the previous work, the dielectric elastomer platform is used to trigger the 2D-to-3D assembly to enable reconfigurable 3D mesostructures for potential applications in radio-frequency circuits with tunable resonant frequency. By contrast, this work presents a kind of bioinspired composite material fabricated by the buckling-guided assembly method and this composite can be combined with smart materials (e.g., dielectric elastomer platforms) for potential applications in artificial muscles and soft robots. Secondly, the previous work did not consider tissue-like, tunable, nonlinear stress-strain responses that represent the key focus of the current work.

## Discussion

In summary, the design concept, fabrication strategies, and quantitive design methods reported in this work provide immediate access to bioinspired soft materials with deterministically tailored, anisotropic mechanical and electrical properties. The approach is applicable to the design and fabrication of a range of soft functional materials with various desirable properties. Demonstrations include successful integrations with several active functionalities (biopotential sensors, bioelectronic stimulators, and dielectric elastomer actuators) and development of electrothermally responsive artificial materials with skeleton muscle-like adaptive mechanical properties. Soft materials with tissue-like mechanical and electrical properties could find broad potential applications in a variety of fields, such as controlled growth of various cell types by providing synergistic guidance cues for tissue engineering^9,14,44^, biointegrated devices^12,52^ with similar mechanical and electrical properties with targeted tissues and organs to blur the biotic-abiotic dissimilarities, and humanoid artificial muscles and soft robots^46^ with human muscle-like mechanical and electrical properties.

## Methods

### Materials and reagents

Ecoflex 00–20, Dragon Skin 10, and Dragon Skin 20 were purchased from Smooth-On. Silbione RT Gel 4717 A/B (Silbione) was purchased from Elkem. PI (Kapton, HN100) was purchased from DuPont. PCL was purchased from Polly Plastics. PEDOT:PSS (1.1 % in H_2_O) was purchased from Sigma–Aldrich. AgNWs (Agnw-40) was purchased from ACS Material. VHB 4910 was purchased from 3 M. Carbon grease was purchased from MG Chemicals. Red dye for marking dots and blue dye for colorizing PCL were purchased from Krylon.

### Fabrication of anisotropic elastomer composites

As shown in Supplementary Fig. 2, the fabrication process of anisotropic elastomer composites starts from the preparation of 2D precursors (PI, PCL, or their combinations) using CO_2_ laser cutting (VLS2.30, Universal Laser System). Next, the 2D precursors are transfer-printed onto prestretched silicone elastomer substrates via selective bonding using silicone adhesives (Sil-poxy, Smooth-On). Releasing the prestrains self-organizes the 2D precursors into 3D skeletons with programmed geometries. Then, pouring encapsulation silicone elastomers onto 3D skeletons, followed by degassing in a vacuum chamber and curing in air. After this, 15 min oxygen plasma treatment (Venus PE25, Plasma Etch Inc.) is used to produce a thin stiff layer (SiO_2_) on the surface of the prestretched sample. Subsequently, conductive materials (PEDOT:PSS, AgNWs, or their combinations) are spray-coated on the sample surface using an airbrush (nozzle size: 0.3 mm; Master Airbrush). Releasing prestrains completes the fabrication of anisotropic elastomer composites. Besides, for the device fabrication in Fig. 3d, a shadow mask is used to guide the patterning of the hybrid of PEDOT:PSS and AgNWs on the sample surface (Supplementary Fig. 27). In addition, for easy visualization of 3D skeletons and their deformations under stretching, Silbione is used as the encapsulation material for the samples shown in Figs. 1c, 2b, 2e and 4d because of its high optical transparency. For these samples, Ecoflex 00–20 is needed as the encapsulation material to achieve the designed mechanical properties.

### Finite element analyses (FEA)

FEA were adopted by employing commercial software ABAQUS (SIMULIA, Providence RI) to analyze post-buckling behaviors of 3D skeletons, as well as deformations and stress-strain responses for elastomer composites. The four-node shell elements and eight-node linear hexahedron hybrid elements were used for the 3D skeletons and elastomers, respectively, and refined meshes ensured the accuracy. For the elastomer solid, a hyperelastic constitutive relation following a Mooney-Rivlin law was adopted in the FEA, noting that a linear elastic constitutive relation can also be adopted when the stress-strain curve of the elastomer meets linear relation approximately to improve the computational efficiency. The material parameters (${C}_{01}$, ${C}_{10}$ and ${D}_{1}$ or $E$ and $\nu$) were determined by fitting the uniaxial stress-strain curve measured in experiments. Both full-scale models and periodic unit segments were exploited in FEA. To save the computational cost based on the periodic unit segments, the displacement components along the uniaxial stretching direction were prescribed, and the boundaries were allowed to deform freely along the transverse direction. The interaction between 3D skeletons and encapsulation materials was taken into account through the ‘embedded interaction’ in ABAQUS.

### Fabrication of dielectric elastomer actuator (DEA) integrated with anisotropic elastomer composites

The fabrication process is illustrated in Supplementary Fig. 20. Briefly stated, the process starts from the preparation of a triple-layer DEA actuator by coating AgNWs and carbon grease on top and bottom sides of the DEA membrane (VHB 4910), respectively. Then, the 2D PI precursors are transfer-printed onto the top side of the prestretched DEA membrane via selective bonding. Here, a commercial adhesive (Super Glue; Gorilla Glue Company) is used to yield strong interfacial bonding at the bonding sites. Subsequently, slightly releasing the prestrain in the DEA membrane to a lower level completes the assembly of 3D skeletons. Finally, the prestretched DEA membrane is fixed on a rigid framework, followed by encapsulation of 3D skeletons using Silbione. For easy visualization of strain distributions, marking dots (diameter: 500 μm; pitch: 5 mm; red dye) are patterned on the surface of the DEA membrane with the aid shadow masks.

### Characterizations and measurements

SEM images were taken with FEI Quanta 600 FEG Environmental SEM. Strain-stress curves were acquired using a Mark-10 ESM303 tensile tester. Sheet resistances were measured using Ossila’s four-point probe system. Infrared images were captured with a commercial thermal camera (FLIR E6). Electrophysiological signals were recorded with PowerLab T26 (AD Instruments). Electrical stimulations were delivered by a digital source meter (Keithley 2604B). Keithley 2230-30-1 was used to provide incident powers for joule-heating elements. A high voltage amplifier (Trek Model 610E) was used to apply actuation voltages in dielectric elastomer actuators. For Fig. 5e, the coordinate positions of red marking dots were processed automatically by the image processing software (PhotoModeler), and the strain distributions were then computed by MATLAB (version 2017b).

### Experiments on human subjects

All experiments were conducted under approval from the Institutional Review Board at the University of Missouri at Columbia (number: 2010272). All human subjects gave written and informed consent before participation in the studies.

## Supplementary information

Supplementary Information

Description for Additional Supplementary Files

Supplementary Movie 1

Supplementary Movie 2

Supplementary Movie 3

## Data Availability

The data that support the findings of this study are available within this article and its Supplementary Information, and from the corresponding authors upon reasonable request.
