# Supplementary material for: Bioinspired elastomer composites with programmed mechanical and electrical anisotropies
Source: Nat Commun. 2022 Jan 26;13:524. doi: 10.1038/s41467-022-28185-z (PMC8791960; doi:10.1038/s41467-022-28185-z)
Supplement: Supplementary file 1 — Supplementary Information [file 41467_2022_28185_MOESM1_ESM.pdf]

Supplementary Information

Bioinspired Elastomer Composites with Programmed Mechanical and Electrical Anisotropies

Yun Ling,[1†] Wenbo Pang,[2,3†] Jianxing Liu,[2,3] Margaret Page,[1] Yadong Xu,[4] Ganggang Zhao,[1] David Stalla,[5] Jingwei Xie,[6] Yihui Zhang, [2,3*] Zheng Yan[1,4*]

[1]Department of Mechanical and Aerospace Engineering, University of Missouri, Columbia, MO 65211, USA

[2]Applied Mechanics Laboratory, Department of Engineering Mechanics, Tsinghua University, Beijing 100084, China

[3]Center for Flexible Electronics Technology, Tsinghua University, Beijing 100084, China

[4]Department of Biomedical, Biological and Chemical Engineering, University of Missouri, Columbia, MO 65211, USA

[5]Electron Microscopy Core, University of Missouri, Columbia, MO 65211, USA

[6]Department of Surgery-Transplant and Mary and Dick Holland Regenerative Medicine Program, College of Medicine, University of Nebraska Medical Center, Omaha, NE 68130, USA

†These authors contribute equally to this work.

[*]Corresponding authors: yihuizhang@tsinghua.edu.cn; yanzheng@missouri.edu

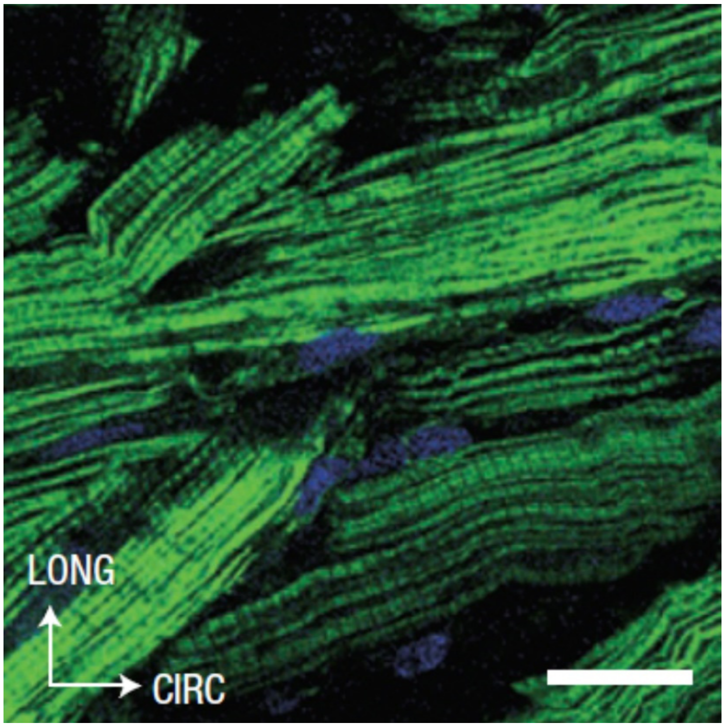

Supplementary Figure 1. A fluorescent image of a rat right ventricular myocardium, showing undulated, preferentially oriented fibrous structures. The circumferential (CIRC) and longitudinal (LONG) axes are labeled. Reproduced with permission from the reference (*Nature Materials* 2008, 7, 1003-1010). Scale bar: 50 µm.

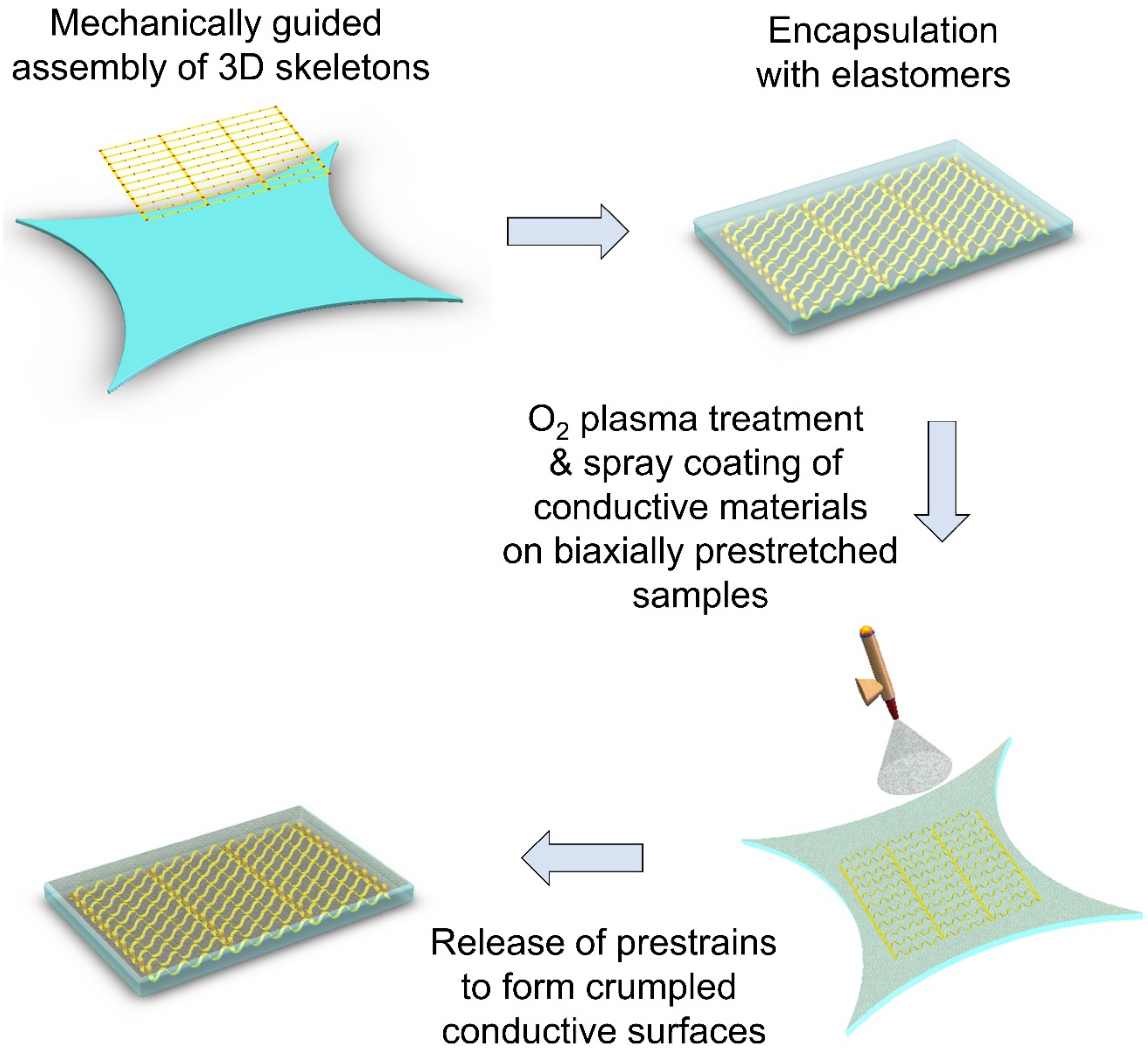

Supplementary Figure 2. A schematic diagram of the fabrication process of bioinspired elastomer composites. Briefly stated, mechanically guided assembly is first used to make 3D plastic (PI, PCL, or their combinations) skeletons with predetermined structures on silicone elastomer supporting substrates, followed by encapsulation of 3D skeletons with silicone elastomers. Next, O_2 plasma treatment is used to induce a thin stiff (SiO_2) layer on the surface of the biaxially prestretched sample. Then, conductive materials (PEDOT:PSS, AgNWs, or their combinations) are coated on the sample surface using spray printing. Finally, releasing prestrains can induce the formation of crumpled conductive surfaces to complete the fabrication of the composite material.

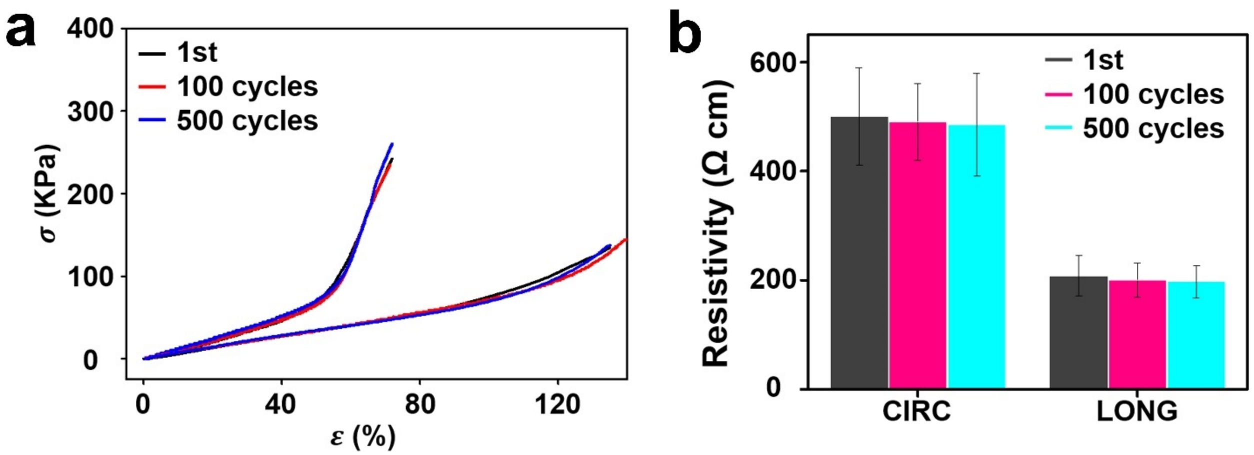

Supplementary Figure 3. Cyclic tests of the obtained heart-like elastomer composites, indicating outstanding endurance of both mechanical **(a)** and electrical **(b)** properties. Here, the electrical cyclic stretching tests are performed with the strains ($\varepsilon_x = 40\%$ and $\varepsilon_y = 150\%$), which are the prestrains used to form crumpled surface structures. Error bars are determined from three samples.

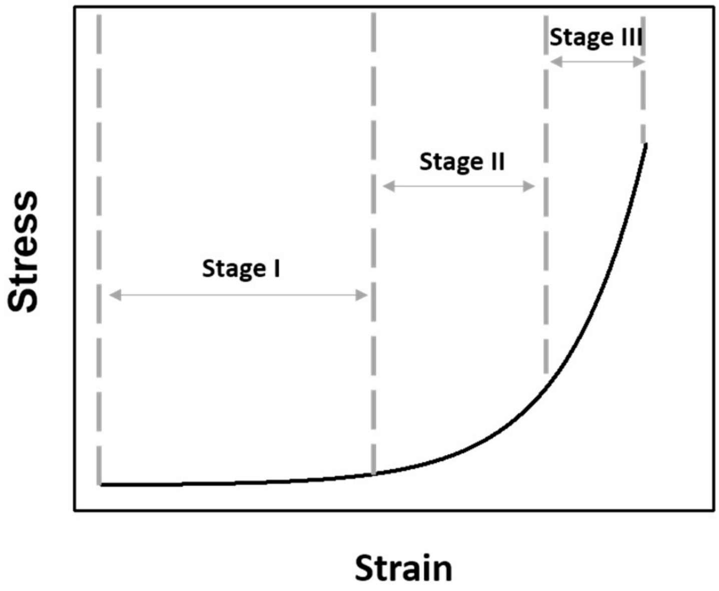

Supplementary Figure 4. Tissue-like, J-shaped, non-linear stress-strain curves are labeled with three major stages.

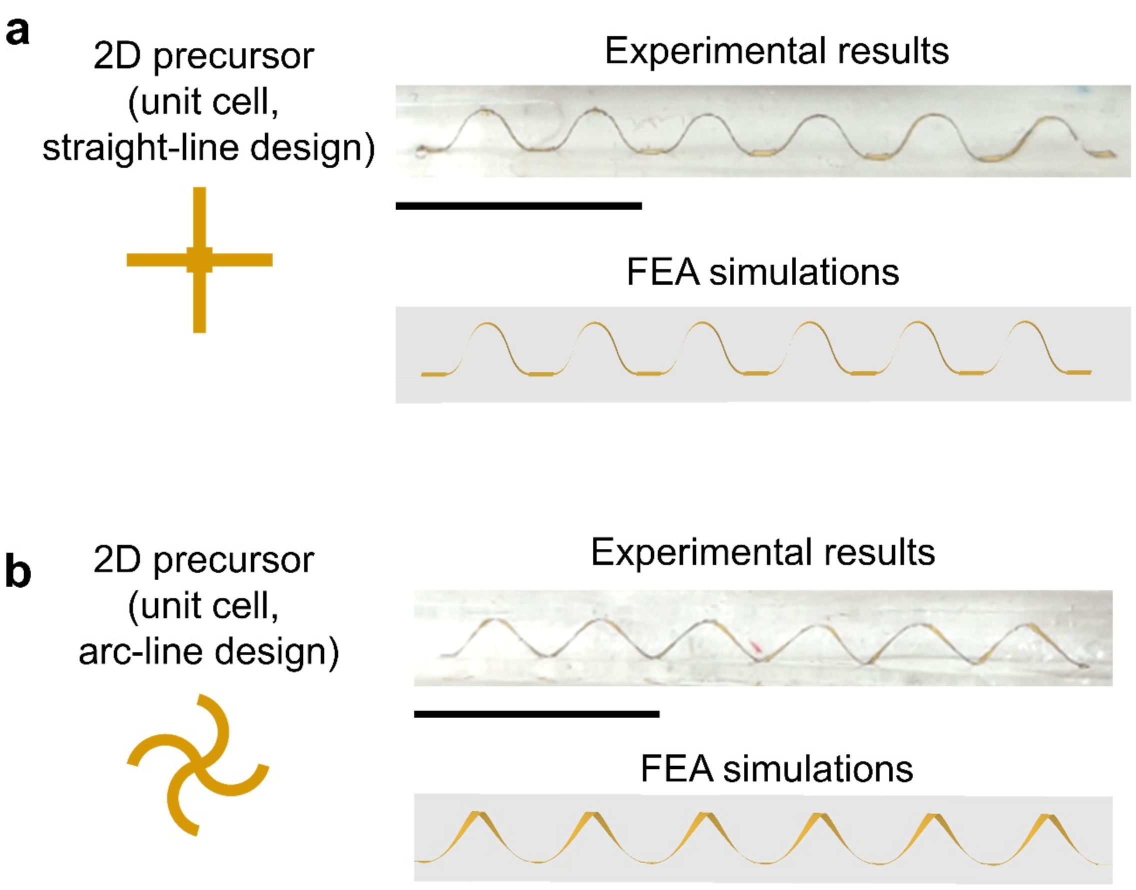

Supplementary Figure 5. Unit cells of 2D precursors, experimental results, and FEA simulations of representative straight-line 3D skeleton designs **(a)** and arc-line 3D skeleton designs **(b).** Here, 2D precursors are shown from the top views. And experimental results and FEA simulations of 3D skeletons are demonstrated from the side views. Scale bars: 1 cm.

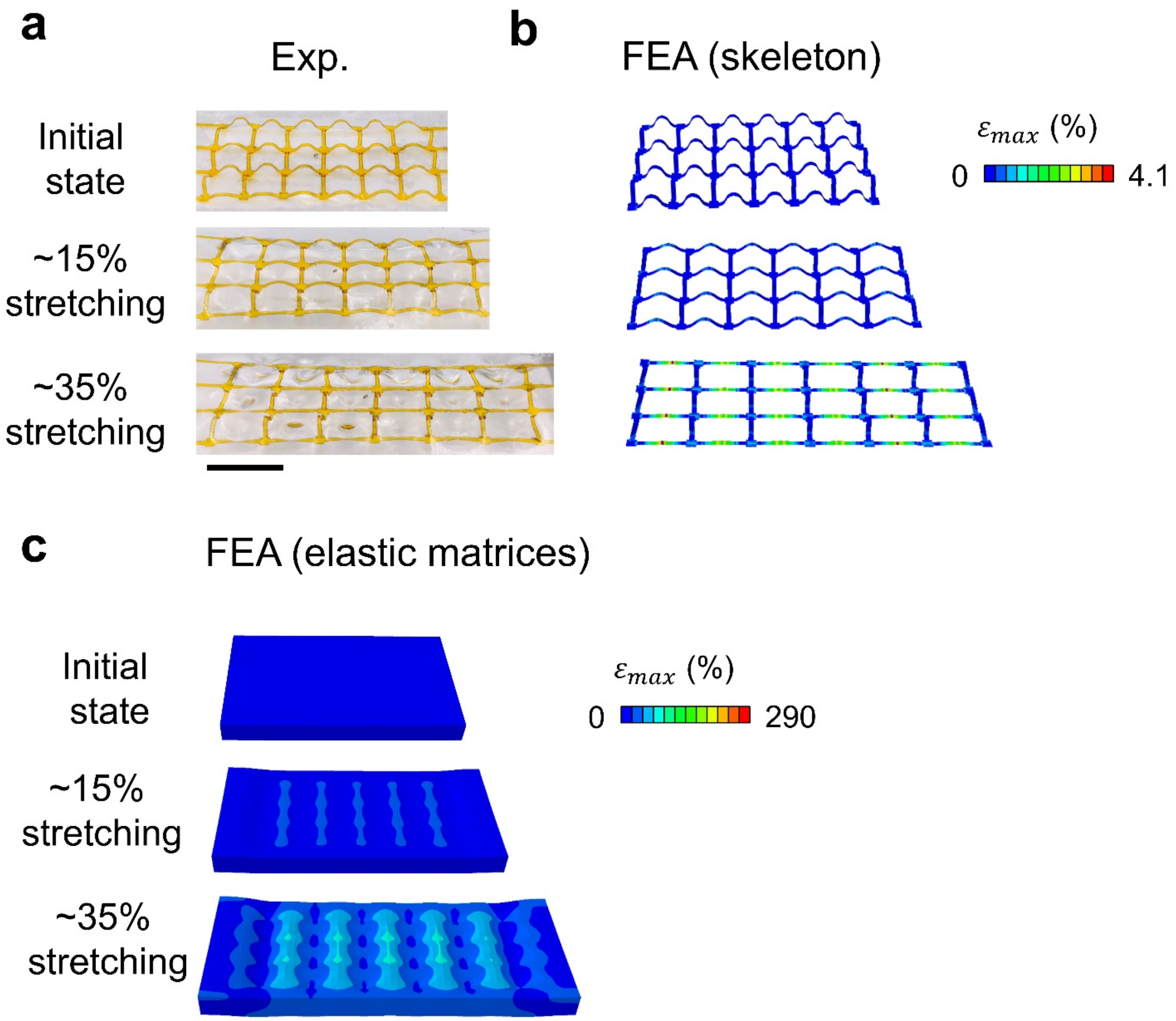

Supplementary Figure 6. Experimental results **(a)**, and FEA simulations of 3D skeletons **(b)** and elastomer matrices **(c)** of one straight-line-based elastomer composite under different levels of uniaxial stretching. Here, color bars indicate maximum principal strain distributions. Scale bar: 1 cm.

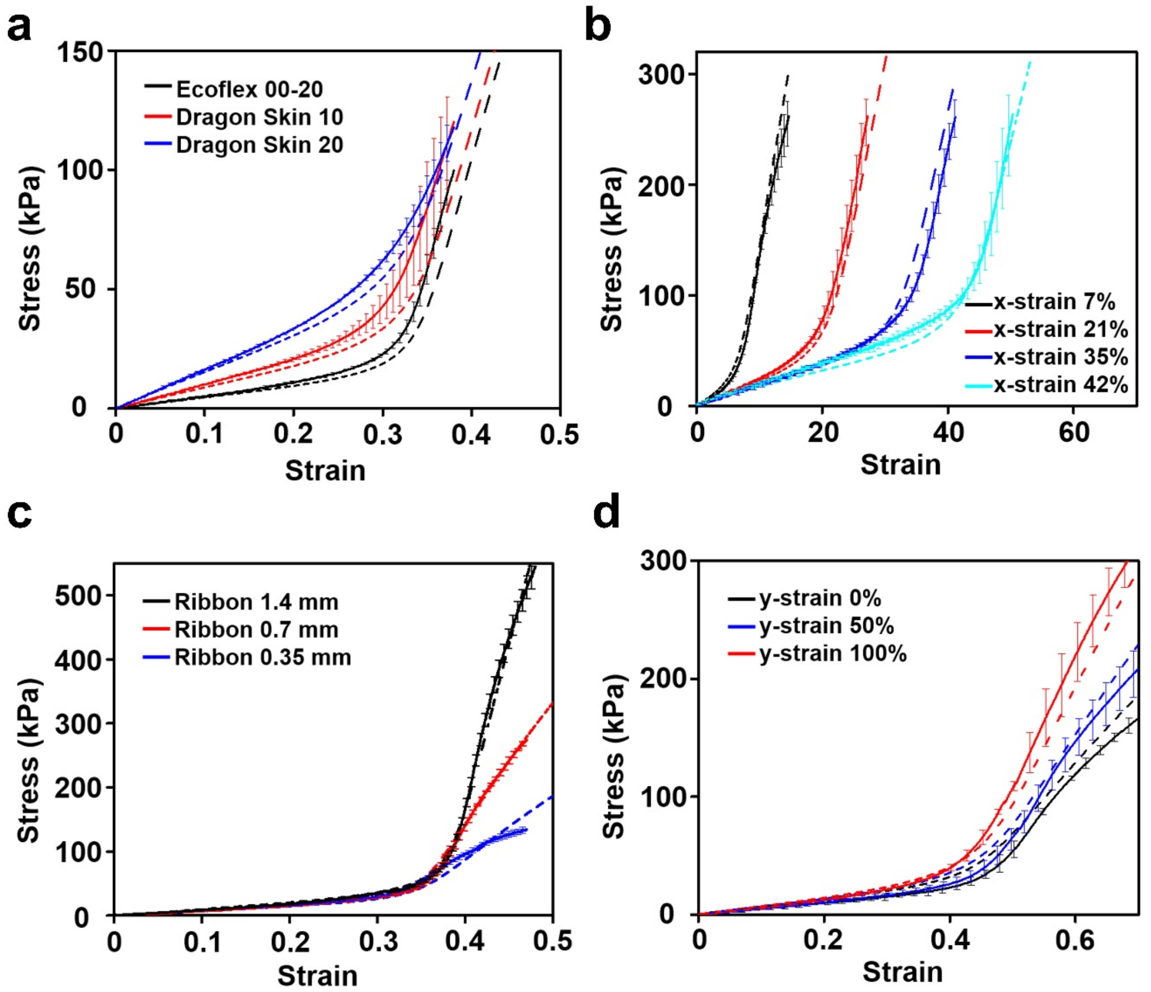

Supplementary Figure 7. Deterministic tailoring of J-shaped stress-strain curves by selections of encapsulation materials **(a)**, applied prestrains in the x axis for 3D assembly **(b)**, ribbon widths **(c)**, and applied prestrains in the y axis for 3D assembly **(d)**, indicating good alignments between the experimental results (solid lines) and FEA simulation results (dash lines). In specific, as shown in **(a)**, the elastic modulus of the encapsulation material can largely dominate the tangential modulus of the elastomer composite in the initial stage (i.e., Stage I). Here, the elastic moduli of Ecoflex 00-20, Dragon Skin 10, and Dragon Skin 20 are ~60 KPa, ~150 KPa, and ~330 KPa, respectively. During the preparation of elastomer composites, the same materials are used for both assembly substrates and encapsulation materials. The critical strain can be tuned by the applied prestrains in the x-axis for 3D assembly **(b)**. The tangential modulus at the critical strain can be tuned by the skeleton ribbon widths **(c)** and applied prestrains in the y-axis for 3D assembly **(d)**. Notably, for the mechanical testing of obtained elastomer composites, the applied uniaxial stretching is along the x-axis. Error bars are determined from three samples.

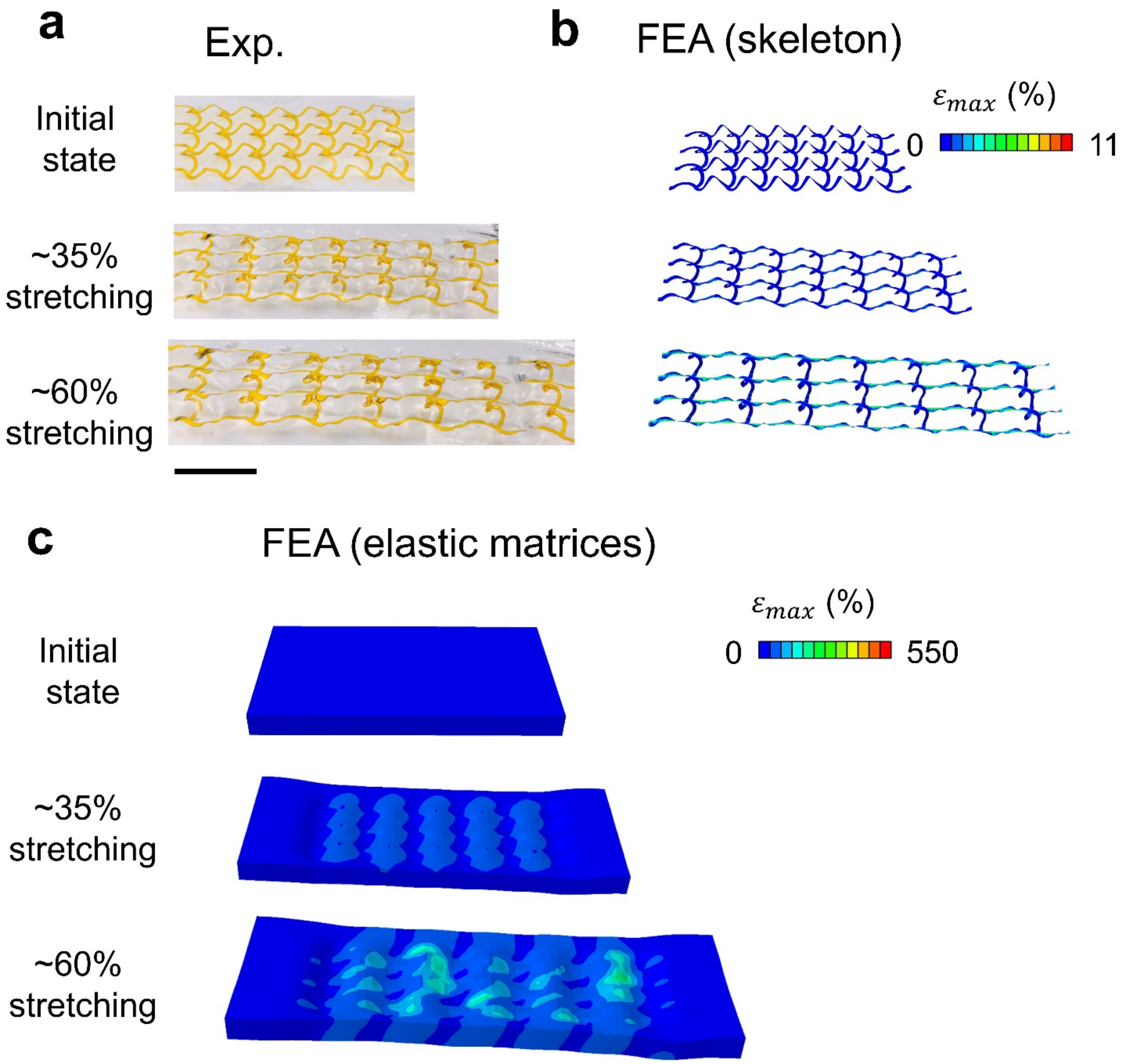

Supplementary Figure 8. Experimental results **(a)**, and FEA simulations of 3D skeletons **(b)** and elastomer matrices **(c)** of one arc-line-based elastomer composite under different levels of uniaxial stretching. Color bars in FEA simulations indicate maximum principal strain distributions. Scale bar: 1 cm.

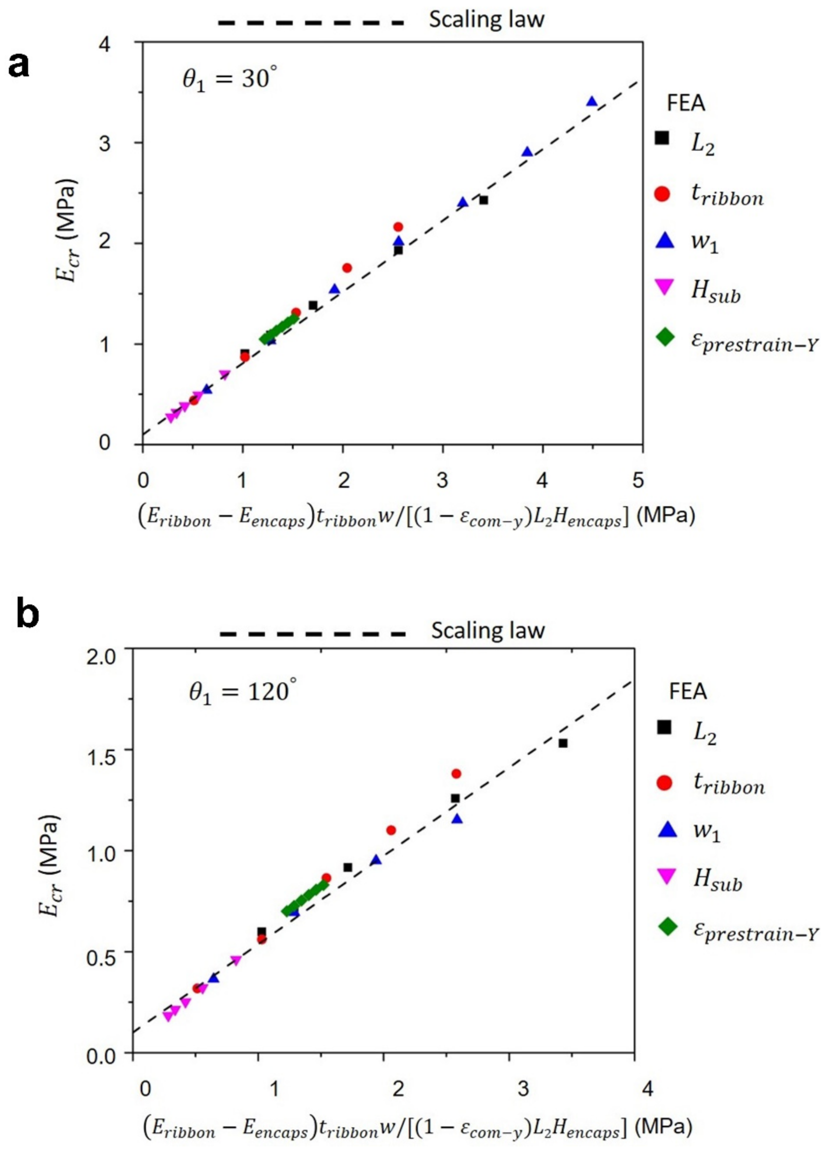

Supplementary Figure 9. The scaling law of E_{cr-arc} versus $(E_{mesh} - E_{elastomer}) \cdot t_{mesh} \cdot w_1 \cdot (1 + \varepsilon_{pre-y})/(L_2 \cdot H_{elastomer})$ for the arc-line design with $\theta_1 = 30°$ **(a)** and $\theta_1 = 120°$ **(b)**.

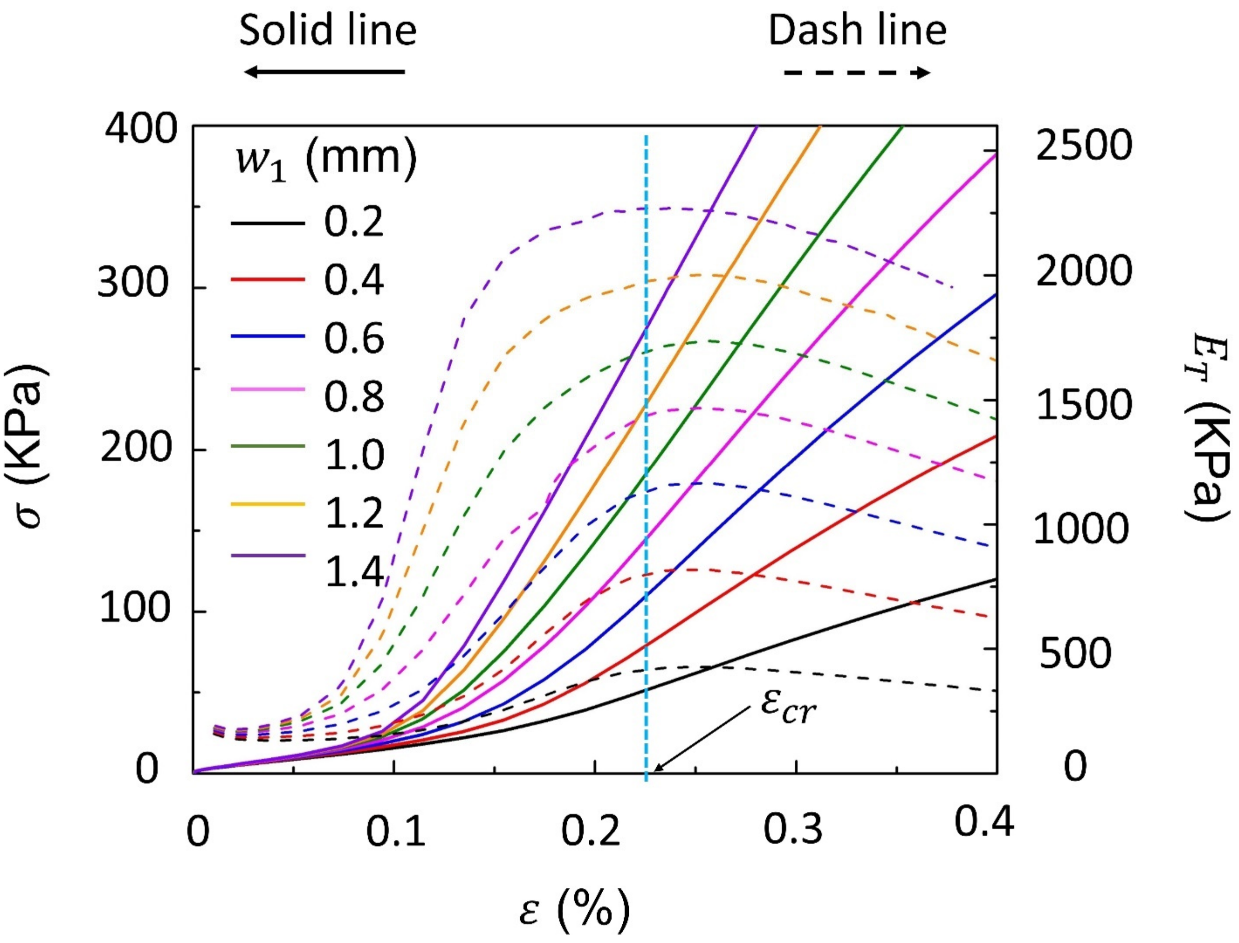

Supplementary Figure 10. The stress-strain curve and tangential modulus for the arc-line design with different ribbon widths. The critical strains for the samples with different ribbon widths are the same, indicating that k_3 is not relative to the ribbon width.

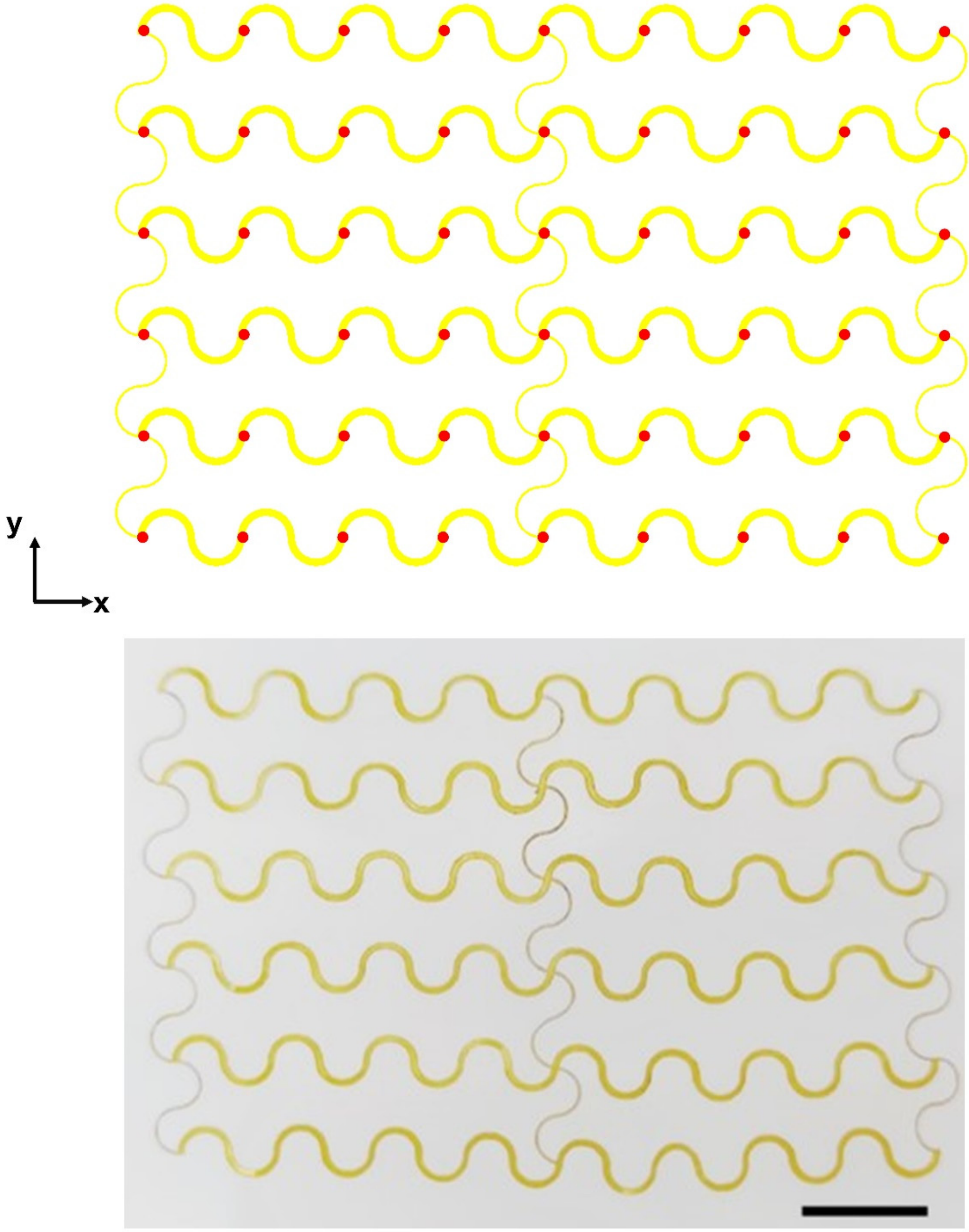

Supplementary Figure 11. The FEA design (top) and experimental result (bottom) of the 2D percussor used for mimicking the myocardium anisotropic mechanical properties. Red dots in the FEA represent bonding sites. Here, the 2D precursor is made of PI with a thickness of 25 µm. The ribbon width along the x-axis is 1.5 mm. And the ribbon width along the y-axis is 0.5 mm. The arc angle is 180°. Scale bar: 1 cm.

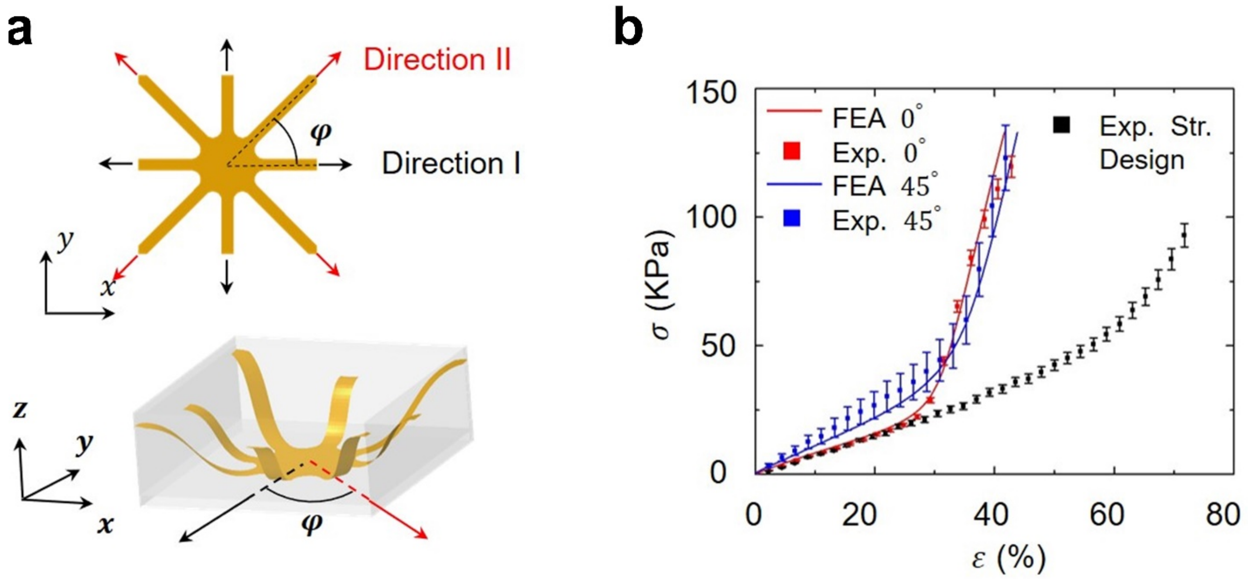

Supplementary Figure 12. a, Schematic illustrations of the unit cell of 2D precursor, 3D skeleton, and elastomer encapsulation for the modified design. **b**, Experimental and FEA results of stress-strain responses for the modified design along x (0°) and diagonal (45°) directions, compared with that for straight-line design (Str. Design) along the diagonal direction. Error bars are determined from three samples.

a

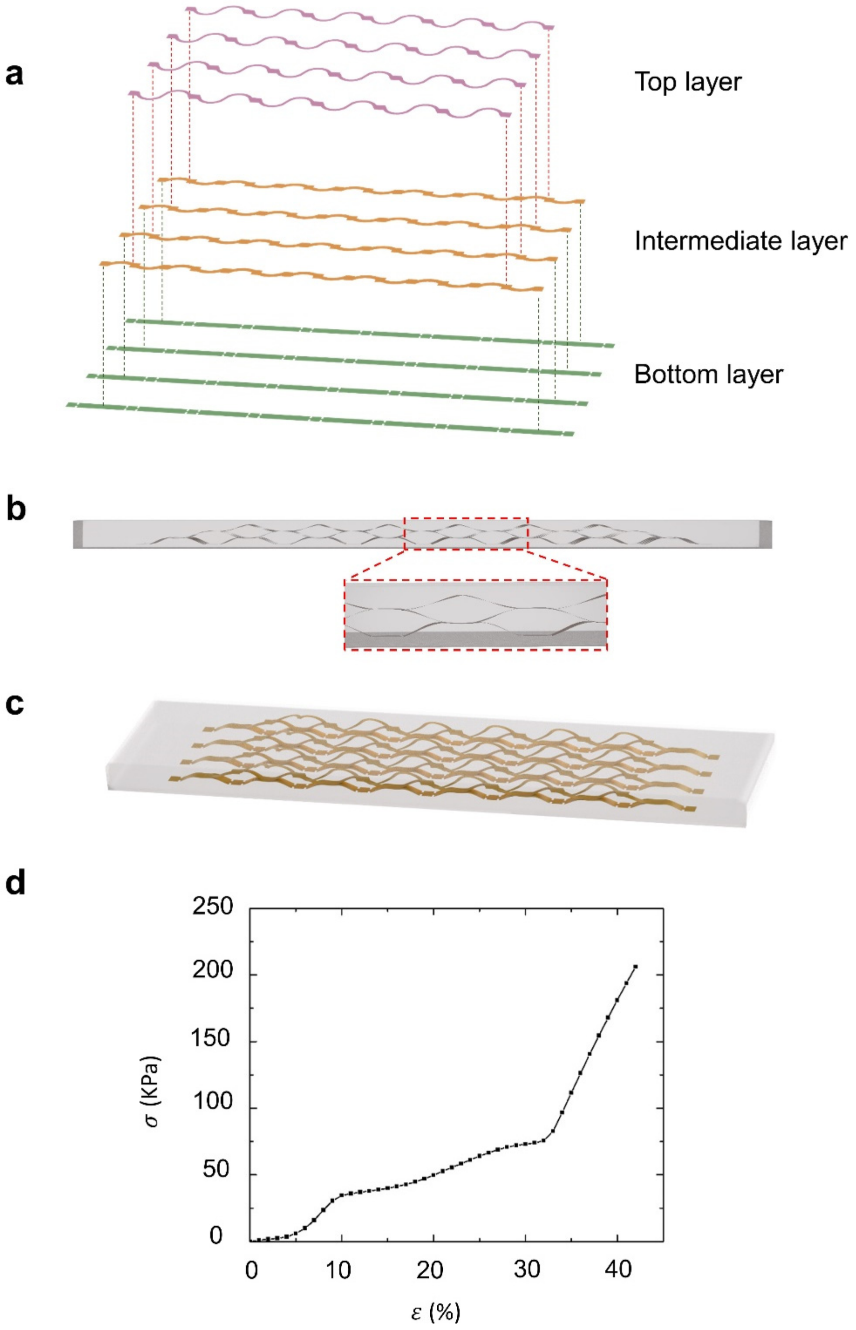

Supplementary Figure 13. a, Exploded view of the 2D precursors for triple-layered 3D skeleton. Schematic illustration of the elastomer composite embedded with triple-layered 3D skeleton from the side view **(b)** and perspective view **(c)**. **d**, Simulated stress-strain responses of the elastomer composite with triple-layered 3D skeleton, indicating multiple critical strains and multi-stage stepped moduli.

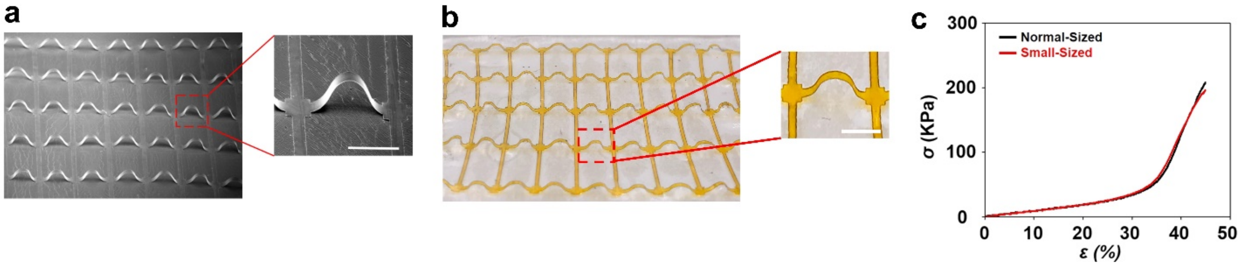

Supplementary Figure 14. a, SEM images of a small-sized 3D skeleton with the unit cell length of ~ 800 µm and the ribbon thickness of ~ 5 µm. Scale bar: 500 µm. **b**, Optical images of a normal-sized 3D skeleton with the unit cell length of ~ 5 mm and the ribbon thickness of ~ 25 µm. Scale bar: 2 mm. **c**, Experimentally measured stress-strain curves of elastomer composites integrated with the normal-sized skeletons (black) and small-sized skeletons (red).

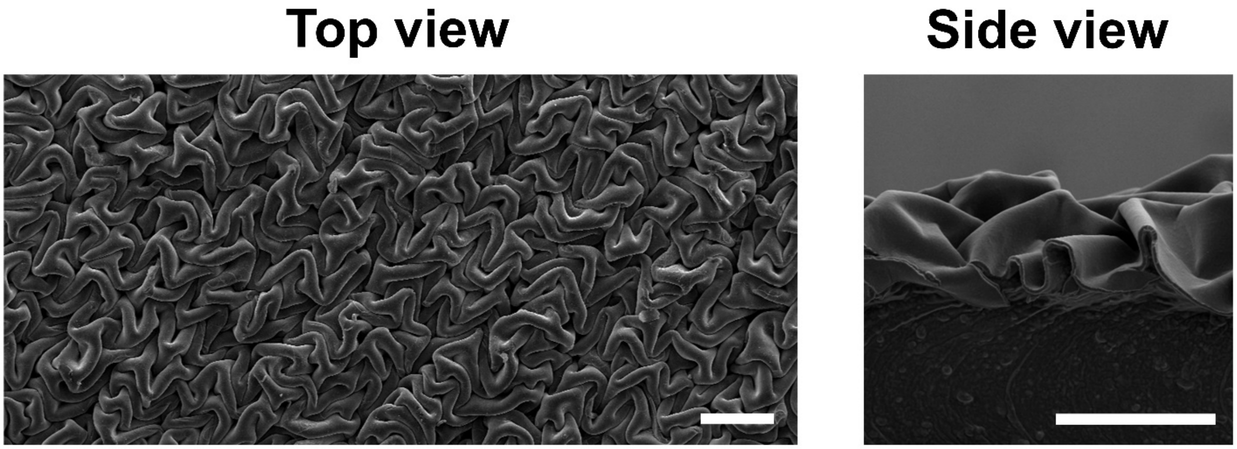

Supplementary Figure 15. SEM images of crumpled anisotropic conductive surfaces formed by applying prestrains of $\varepsilon_x = 100\%$ and $\varepsilon_y = 100\%$. Here, PEDOT:PSS is coated as the surface conductive layer. Scale bars: 100 μm.

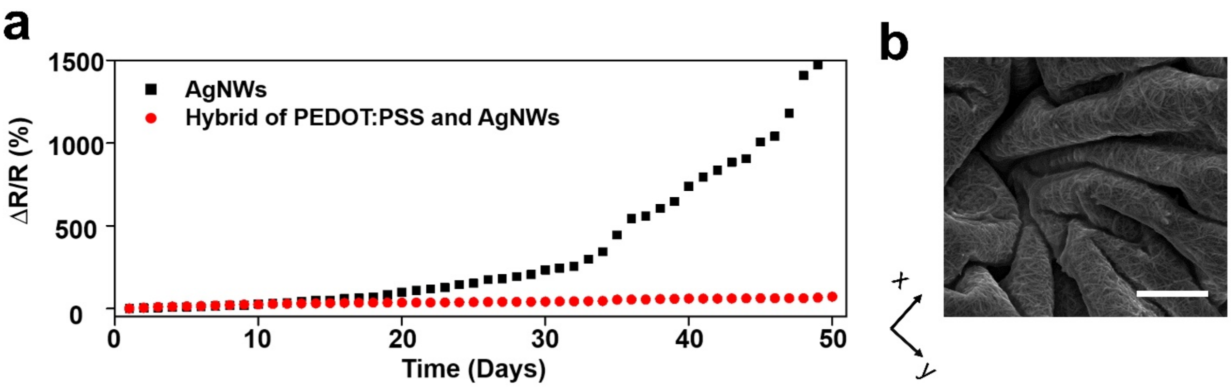

Supplementary Figure 16. a, Resistance changes of crumped conductive surfaces coated with AgNWs and hybrids of PEDOT:PSS and AgNWs as a function of time exposed to air. After exposure to air for 50 days, the resistance of the sample coated with AgNWs increased about 1500% and the sample coated with the hybrids increases about 70%. Their initial sheet resistances are ~15 Ω/□. **b**, SEM image of crumpled anisotropic conductive surfaces formed by applying prestrains of $\varepsilon_x = 50\%$ and $\varepsilon_y = 150\%$. Here, the hybrids of PEDOT: PSS and AgNWs are coated as surface conductive layers. Scale bar: 100 μm.

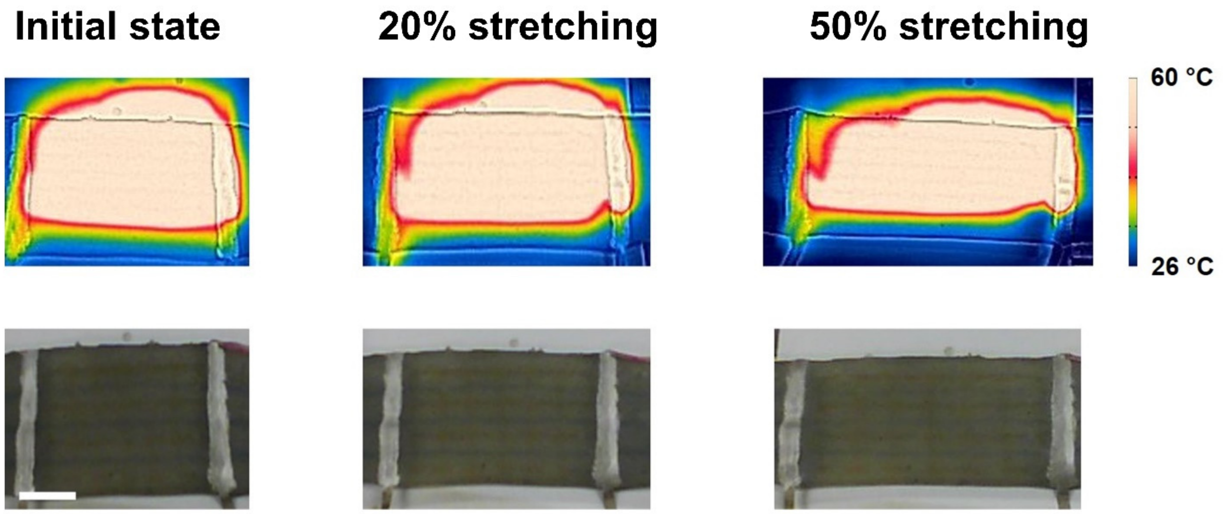

Supplementary Figure 17. Infrared images (top) and optical images (bottom) of the joule heater at the initial state, 20% stretching, and 50% stretching. Scale bar: 1 cm.

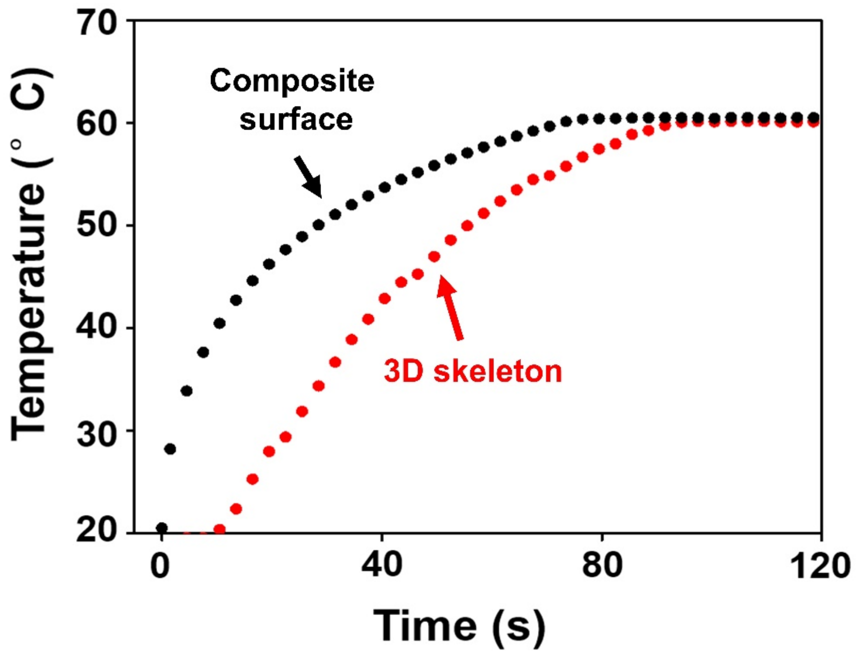

Supplementary Figure 18. Simultaneous measurements of temperature changes of the composite surfaces and 3D skeleton during heating process.

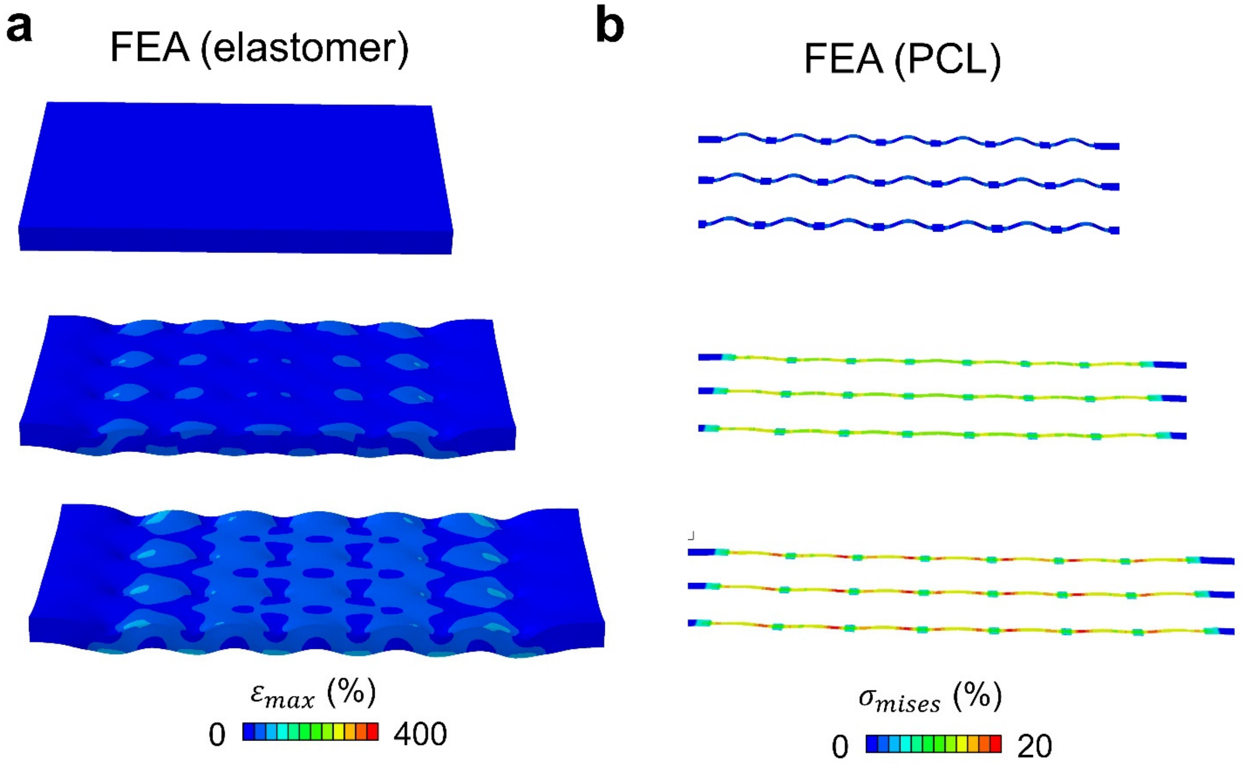

Supplementary Figure 19. The FEA simulations of maximum principal strain distributions of the encapsulation material and the Mises stress distributions of PCL skeletons for the PCL/PI hybrid elastomer composite at different levels of tensile strains (0%, 12%, and 28% from top to bottom).

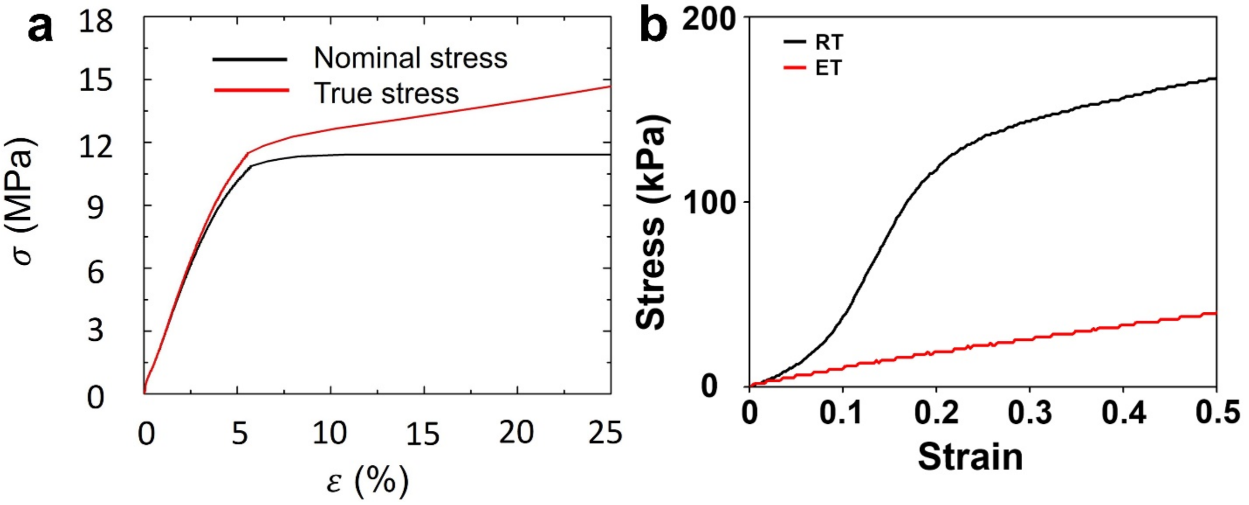

Supplementary Figure 20. a, The stress-strain curve for the PCL without patterns under uniaxial stretching, indicating a typical elastic-plastic behavior. Redline represents true stress versus stretching strain responses, and the black line represents nominal stress versus stretching strain responses. **b**, The stress-strain curves of the elastomer composite embedded with 3D PCL skeletons at room temperature (RT; black curve) and elevated temperature (ET; ~60ºC; red curve). When PCL skeletons melt, the composite exhibits the same mechanical behavior as the encapsulation elastomer.

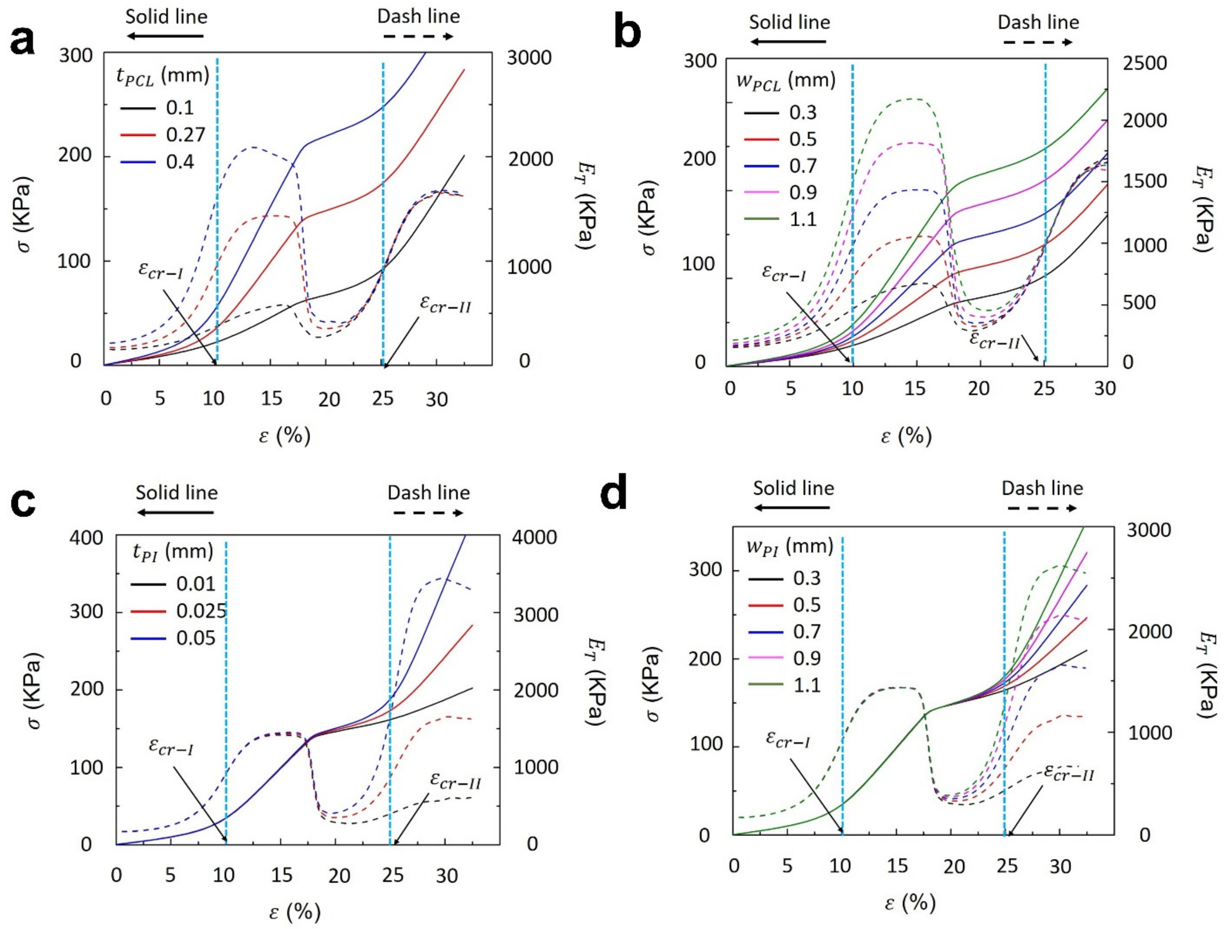

Supplementary Figure 21. The FEA simulations of the stress-strain curves and tangential moduli of the PCL/PI hybrid elastomer composites with various t_{PCL} **(a)**, w_{PCL} **(b)**, t_{PI} **(c)**, and w_{PI} **(d)** at room temperature.

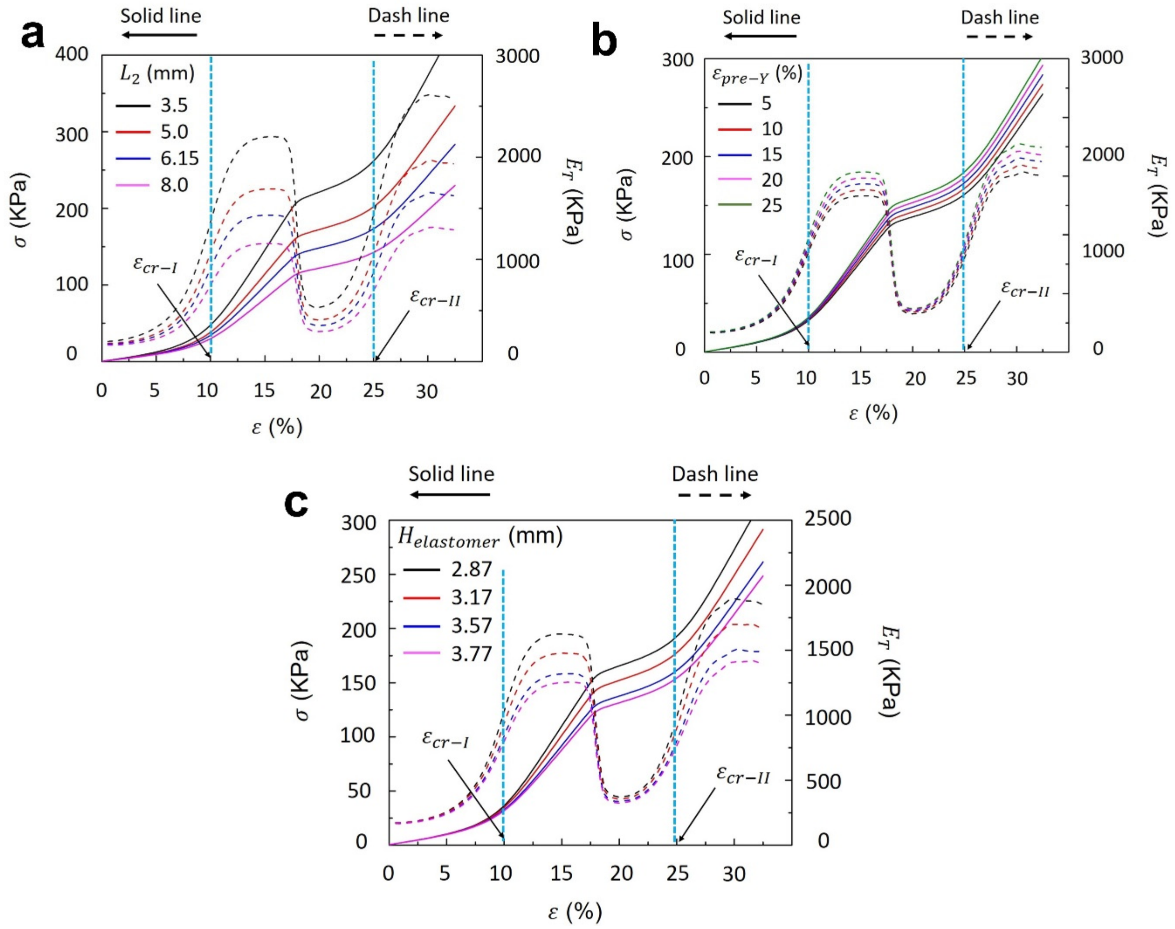

Supplementary Figure 22. The FEA simulations of the stress-strain curves and tangential moduli of the PCL/PI hybrid elastomer composites with various L_2 **(a)**, ε_{pre-Y} **(b)**, $H_{elastomer}$ **(c)** at room temperature.

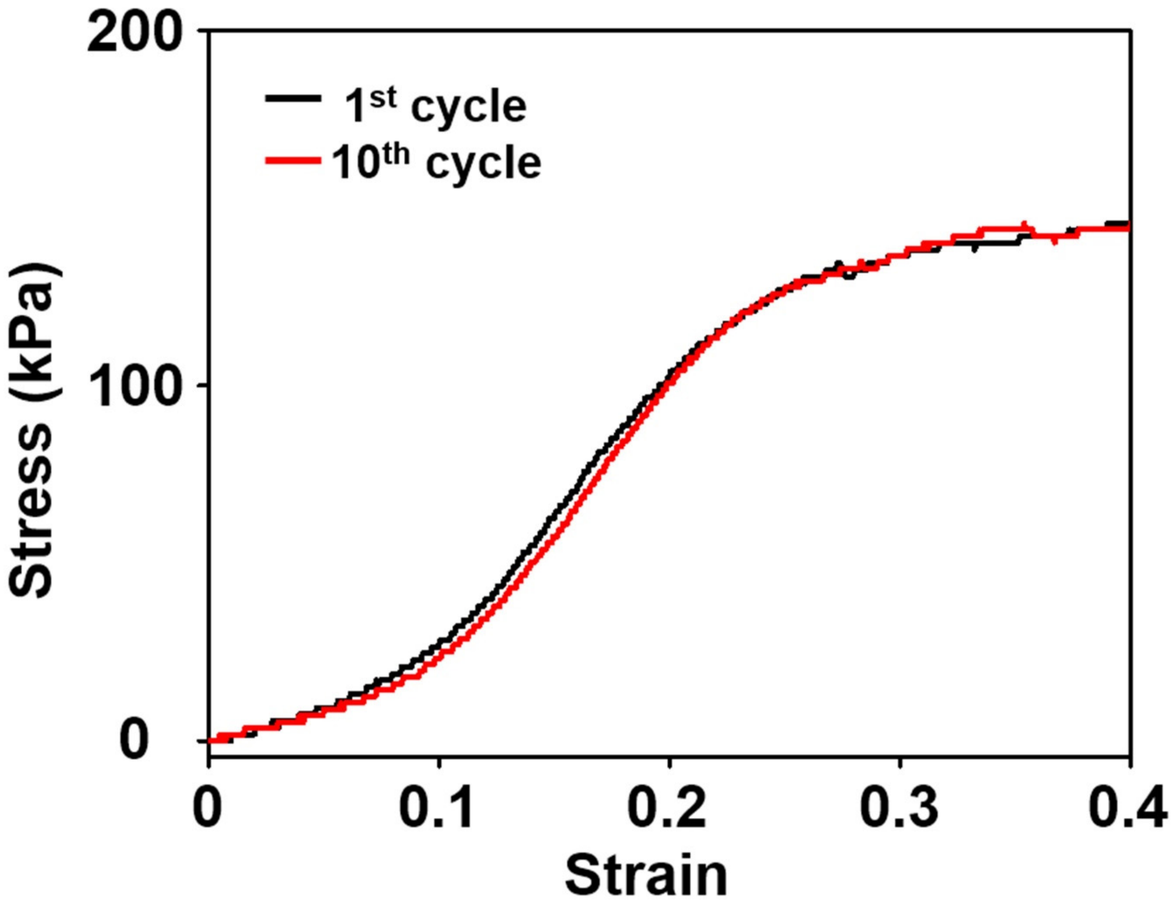

Supplementary Figure 23. Cyclic testing of the stress-strain curve of the elastomer composite embedded with the 3D PCL skeleton at room temperature. As demonstrated in this figure, the overstretched (i.e., stretched to the plastic stage) PCL skeleton can be restored to the initial state by releasing the applied prestrain (~40%) at the elevated temperature (~60°C) and then cooling down the sample to room temperature.

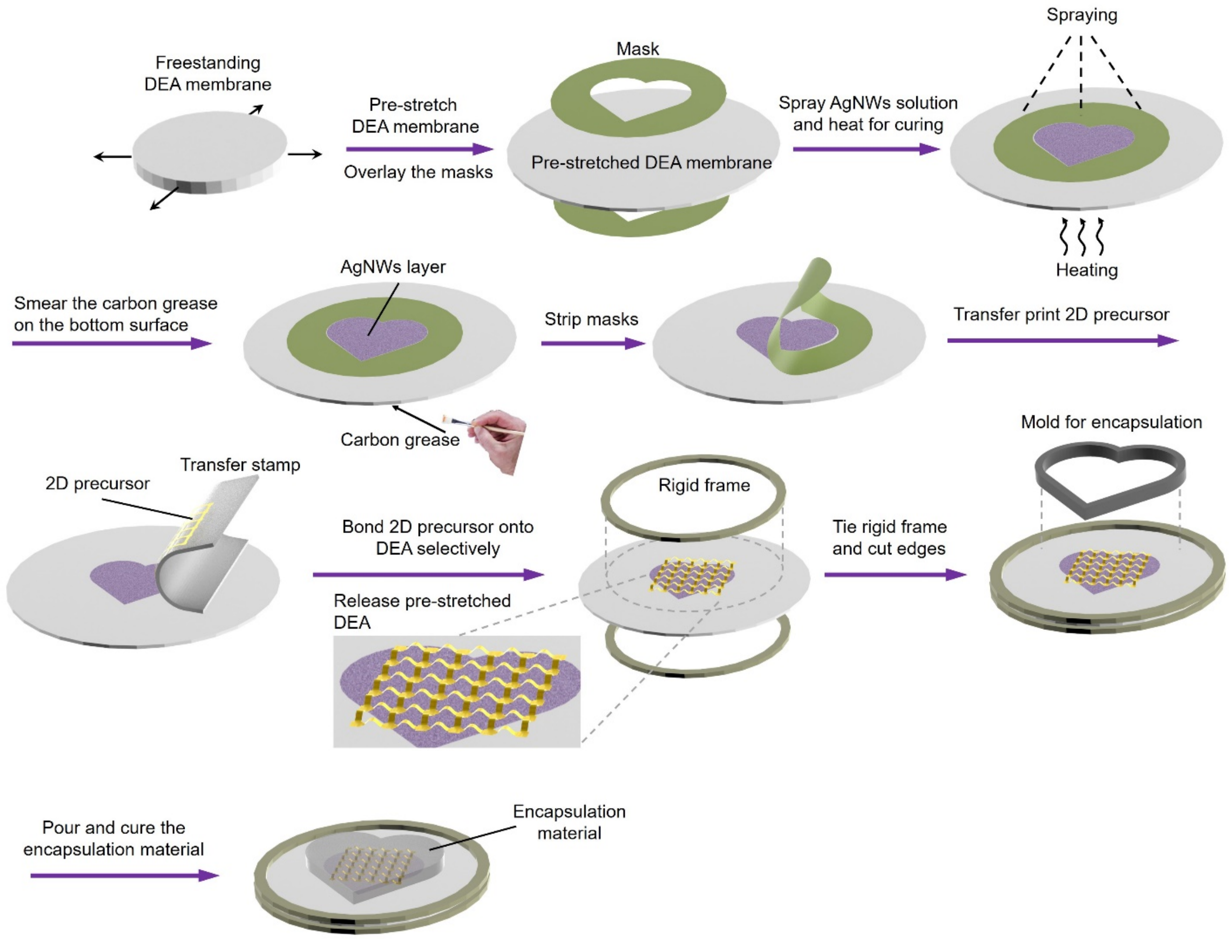

Supplementary Figure 24. Schematic illustrations of the fabrication process of a heart-like dielectric elastomer actuator (DEA) integrated with anisotropic elastomer composites.

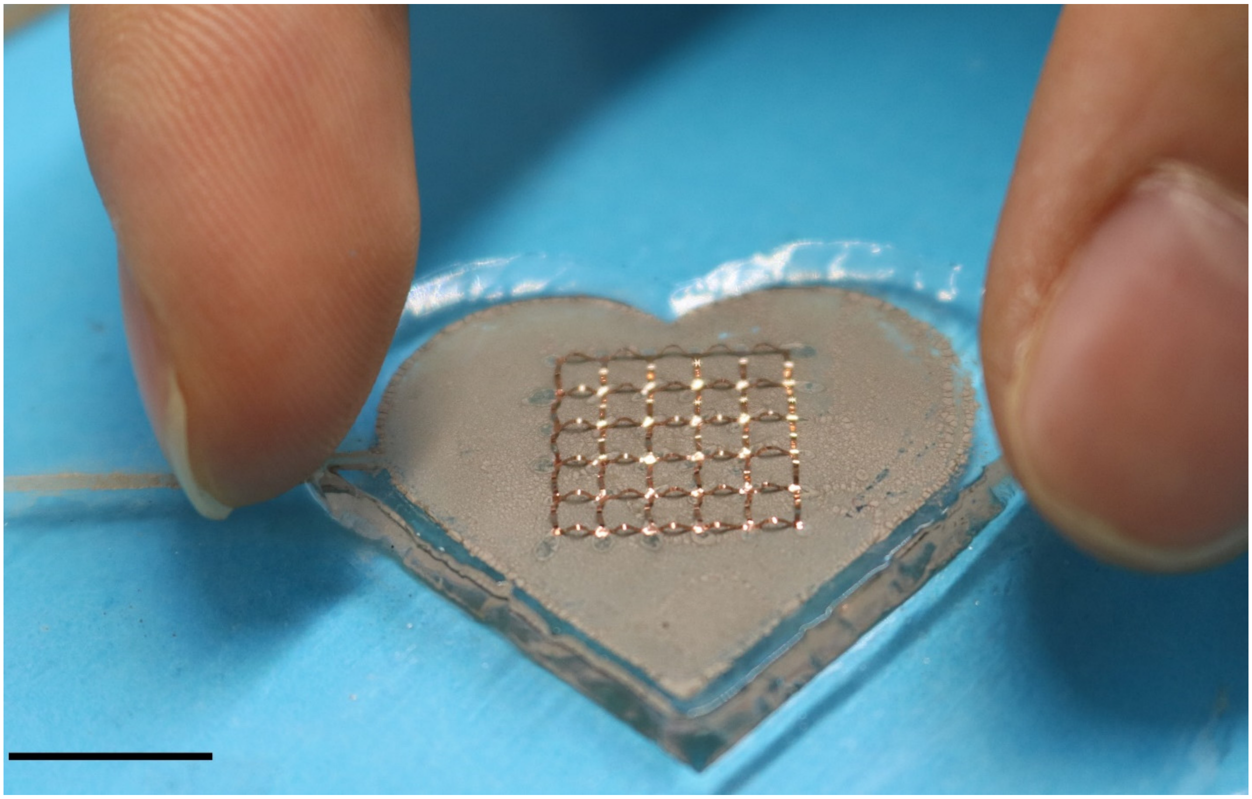

Supplementary Figure 25. Optical image of a heart-like dielectric elastomer actuator integrated with anisotropic elastomer composites. Scale bar: 1 cm.

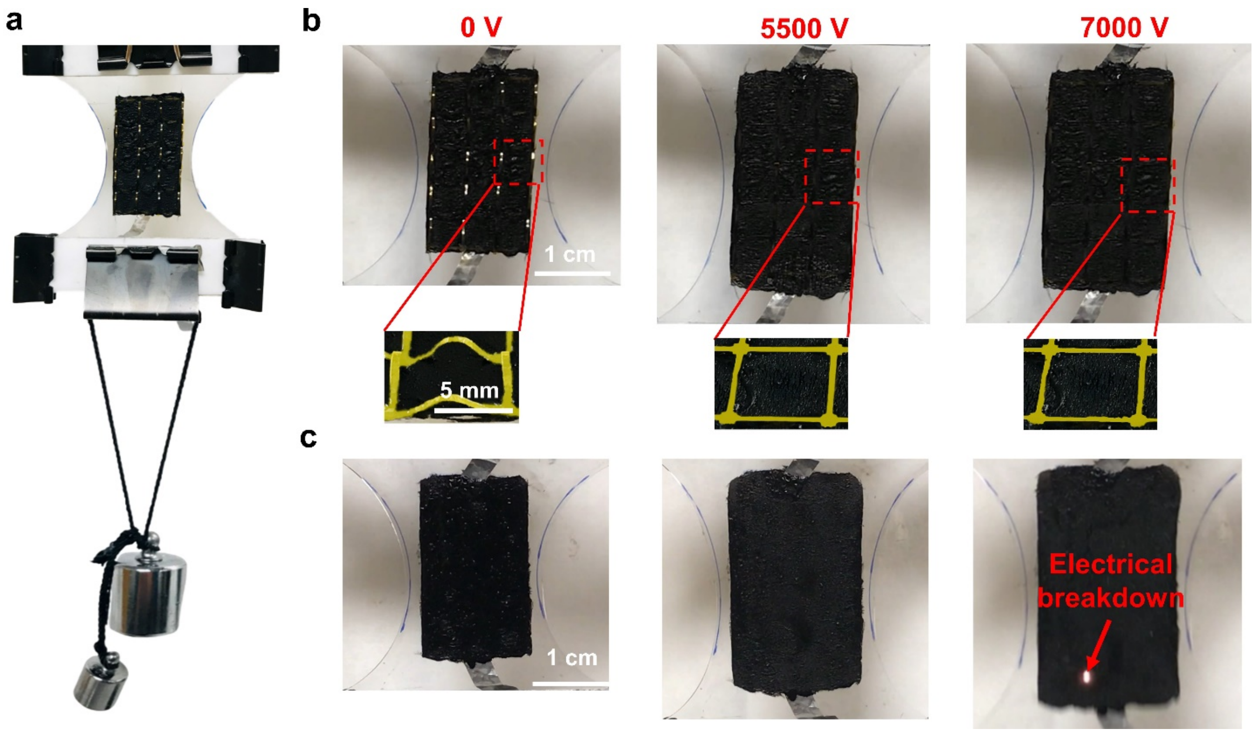

Supplementary Figure 26. a, Optical image of DEA-based artificial muscle with 3D skeleton that can be used to lift weights. Magnified optical images of DEA-based artificial muscles with **(b)** and without **(c)** 3D skeletons at different actuation voltages (from left to right: 0 V, 5500 V and 7000 V). Insets are the colorized optical images of 3D skeletons at different actuation voltages.

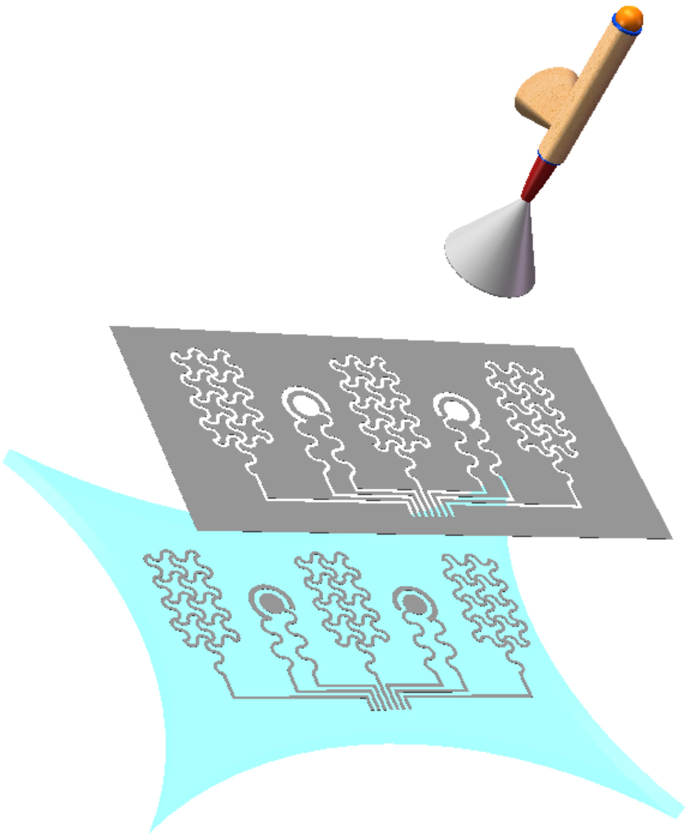

Supplementary Figure 27. Schematic illustrations of spray printing of bioelectronic sensors and stimulators, shown in Fig. 4d, on anisotropic elastomer composites.
