# Supplementary material for: Bioinspired elastomer composites with programmed mechanical and electrical anisotropies
Source: Nat Commun. 2022 Jan 26;13:524. doi: 10.1038/s41467-022-28185-z (PMC8791960; doi:10.1038/s41467-022-28185-z)
Supplement: Supplementary file 2 — Description for Additional Supplementary Files [file 41467_2022_28185_MOESM2_ESM.pdf]

File Name: Supplementary Movie 1

Description: Anisotropic Elastomer Composites Integrated with Heart-like Dielectric Elastomer Actuators

File Name: Supplementary Movie 2

Description: Dielectric Elastomer Actuators-Based Artificial Muscles without 3D Skeletons

File Name: Supplementary Movie 3

Description: Dielectric Elastomer Actuators-Based Artificial Muscles with 3D Skeletons
